# A Novel Calcium Uptake Transporter of Uncharacterized P-Type ATPase Family Supplies Calcium for Cell Surface Integrity in *Mycobacterium smegmatis*

**DOI:** 10.1128/mBio.01388-17

**Published:** 2017-09-26

**Authors:** Hemant Kumar Gupta, Shruti Shrivastava, Rakesh Sharma

**Affiliations:** a CSIR-Institute of Genomics and Integrative Biology, Council of Scientific and Industrial Research (CSIR), New Delhi, India; b Academy of Scientific and Industrial Research (AcSIR), New Delhi, India; PHRI-Rutgers; Harvard School of Public Health

**Keywords:** calcium, *Mycobacterium smegmatis*, *Mycobacterium tuberculosis*, P-type ATPase, transporter

## Abstract

Ca^2+^ plays an important role in the physiology of bacteria. Intracellular Ca^2+^ concentrations are tightly maintained in the nanomolar range. Molecular mechanisms of Ca^2+^ uptake in bacteria remain elusive. Here we show that CtpE is responsible for Ca^2+^ uptake in *Mycobacterium smegmatis*. It represents a previously uncharacterized P-type ATPase family in bacteria. Disruption of *ctpE* in *M. smegmatis* resulted in a mutant with impaired growth under Ca^2+^-deficient conditions. The growth defect of the mutant could be rescued by Ca^2+^ or by ectopic expression of *ctpE* from *M. smegmatis* or the orthologous gene (*Rv0908*) from *Mycobacterium tuberculosis* H37Rv. Radioactive transport assays revealed that CtpE is a Ca^2+^-specific transporter. Ca^2+^ deficiency increased expression of *ctpE*, resulting in increased ^45^Ca^2+^ accumulation in cells. *ctpE* is a gene that is part of an operon, which is negatively regulated by Ca^2+^. The *ctpE* mutant also showed hypersensitivity to polymyxin B, increased biofilm formation, and higher cell aggregation, indicating cell envelope defects. Our work establishes, for the first time, the presence of Ca^2+^ uptake pumps of the energy-dependent P-type ATPase superfamily in bacteria and also implicates that intracellular Ca^2+^ is essential for growth and cell envelope integrity in *M. smegmatis*.

## IMPORTANCE

Ca^2+^ is essential for gene regulation, enzymatic activity, and maintenance of structural integrity of cell walls in bacteria. Bacteria maintain intracellular calcium concentrations in a narrow range, creating a gradient with low cytoplasmic calcium concentration and high extracellular calcium concentration. Due to this steep gradient, active pumps belonging to family 2 of P-type ATPases and antiporters are used for Ca^2+^ efflux, whereas Ca^2+^ uptake is usually carried out by channels. Molecular mechanisms of Ca^2+^ uptake in bacteria are still elusive and are mainly limited to a nonproteinaceous channel in *Escherichia coli* and a pH-dependent channel protein from *Bacillus subtilis*. Energy-dependent active transporters are not reported for Ca^2+^ uptake from any organism. Here we show that CtpE belonging to a family of previously uncharacterized bacterial P-type ATPases is involved in specific uptake of Ca^2+^ into *Mycobacterium smegmatis*. We also demonstrate that intracellular Ca^2+^ obtained through CtpE is essential for growth and maintenance of cell surface properties under Ca^2+^-deficient conditions.

## INTRODUCTION

Calcium (Ca^2+^) plays an important role in the physiology of living organisms. These functions have been well characterized for eukaryotes and have begun to be characterized in prokaryotes (1). In prokaryotes, calcium has been implicated in chemotaxis (2), heterocyst differentiation (3, 4), twitching (5) and swarming (6) motility, gene regulation (7), virulence (8, 9), and survival in an eukaryotic host (10). Another important function of Ca^2+^ is to maintain structural integrity of the bacterial membranes and cell wall (11). These functions are mediated by Ca^2+^, either by acting as a structural or catalytic cofactor for various enzymes (12, 13) or by interaction of various Ca^2+^-binding proteins (3, 5, 14, 15).

Ca^2+^ concentration is tightly maintained in bacteria, usually many folds lower than the surrounding environment (1, 16, 17). Ca^2+^ homeostasis requires orchestrated activity of efflux and uptake systems along with Ca^2+^-binding proteins. Ca^2+^ efflux requires transport against a stiff concentration gradient. Active transporters belonging to P-type ATPases of family 2 and antiporters have been identified from bacteria for their role in Ca^2+^ efflux (10, 18–21). Examples of molecular mechanisms of Ca^2+^ uptake in bacteria are few and limited to Ca^2+^ channels, *viz*., a nonproteinaceous Ca^2+^-selective channel from *Escherichia coli* (22) and a recently identified pH-sensitive Ca^2+^ channel from *Bacillus subtilis* (23). Though energy-dependent Ca^2+^ uptake is expected to be essential for survival in Ca^2+^-deficient situations, to the best of our knowledge, to date, no active transporter has been identified and characterized from any organism.

P-type ATPases form a large superfamily of integral membrane proteins involved mainly in ATP-dependent transport of cations and phospholipids (24). Nine functionally characterized P-type ATPase families have been identified and are further classified into three groups; topological type 1 with 8 transmembrane α-helical segments (TMS) represents heavy metal ATPases, topological type II with 10 TMS represents the Na^+^/K^+^, Ca^2+^, H^+^, Mg^2+^, Na^+^, K^+^, or phospholipid ATPases, and topological type III represents 4 component K^+^ transporters (25). Genome sequencing has revealed previously uncharacterized P-type ATPases from eukaryotes as well as prokaryotes (25, 26). A comparative study on bacterial P-type ATPases has identified 10 new P-type ATPase families of unknown physiological functions (26). Out of these 10 new families, two families (families 23 and 24) clustered loosely together with family 2 Ca^2+^-ATPases (26) and belong to topology type II, indicating their possible involvement in Ca^2+^ transport. In this study, we selected CtpE, a member of family 23, from *Mycobacterium smegmatis* for further characterization. Here we present evidence that CtpE is a Ca^2+^-specific uptake transporter in mycobacteria. It is expressed as part of an operon, and expression of this operon was found to be negatively regulated by Ca^2+^. We further demonstrate that under conditions of Ca^2+^ deficiency, CtpE supplies Ca^2+^ for growth and maintenance of cell surface integrity.

## RESULTS

### Disruption of *ctpE* results in a mutant sensitive to Ca^2+^ chelators.

**FIG 1:**
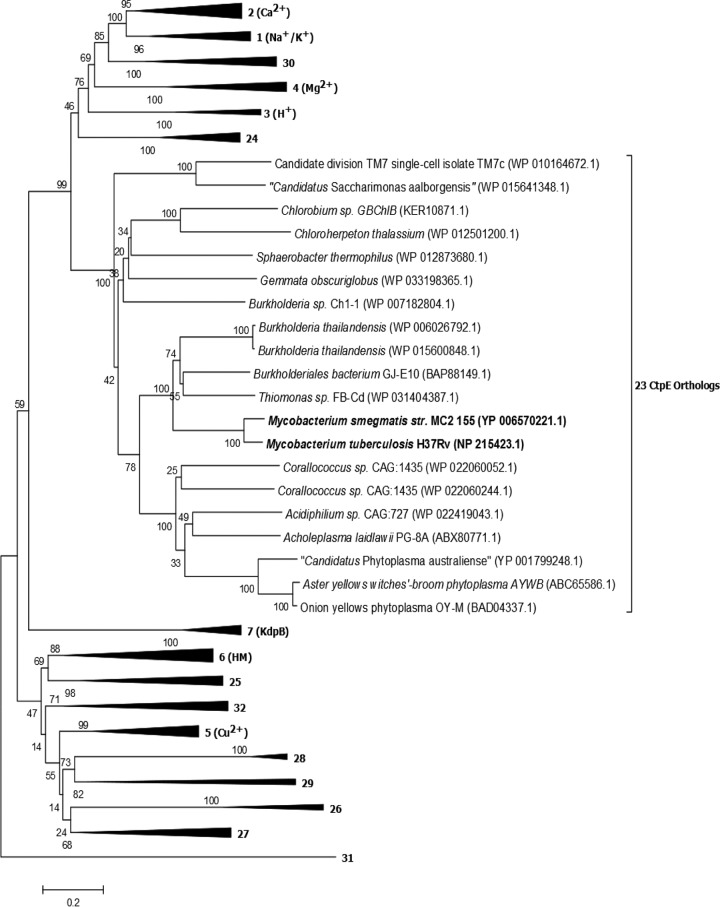
Phylogenetic tree depicting P-type ATPases families across bacterial divisions. The boldface numbers after the black triangles or line give the P-type ATPase family numbers; substrates for the characterized families are shown after the family number in parentheses. CtpE orthologs from “*Candidatus* Saccharibacteria,” *Chlorobi*, *Chloroflexi*, *Planctomycetes*, *Proteobacteria*, and *Tenericutes* that were not previously reported are shown along with CtpE from *Mycobacterium smegmatis* and *Mycobacterium tuberculosis*. The accession numbers for CtpE orthologs are shown in parentheses. The accession numbers of the sequences representing other families are provided in Text S1 in the supplemental material. The bar indicates 0.2 nucleotide substitutions per position.

*Mycobacterium smegmatis* CtpE belongs to family 23 of functionally uncharacterized P-type ATPases of bacteria (26). We observed that in addition to the reported members from *Actinobacteria*, *Firmicutes*, and *Cyanobacteria* for this uncharacterized family, orthologs were also present in “*Candidatus* Saccharibacteria,” *Chlorobi*, *Chloroflexi*, *Planctomycetes*, *Proteobacteria*, and *Tenericutes* (Fig. 1 and see Table S1 in the supplemental material). *M. smegmatis ctpE* encodes a putative P-type ATPase of 791 amino acids. It has 10 predicted transmembrane helices (TM) and 2 cytoplasmic loops after TM2 and TM4 (Fig. S1). It has all the conserved motifs for functional activity that P-type ATPase has (Fig. S1). A conserved motif VPEGL was observed in TM4, which is similar to the IPEG(A)L motif observed in Ca^2+^-transporting P-type ATPases of family 2, indicating that Ca^2+^ may be a probable substrate for family 23.

10.1128/mBio.01388-17.2 FIG S1  Schematic depicting the predicted topology and conserved residues of the CtpE protein. Download FIG S1, TIF file, 8.2 MB. Copyright © 2017 Gupta et al. 2017 Gupta et al. This content is distributed under the terms of the Creative Commons Attribution 4.0 International license.

10.1128/mBio.01388-17.6 TABLE S1  Orthologs of CtpE identified from various bacterial genomes. Download TABLE S1, DOCX file, 0.1 MB. Copyright © 2017 Gupta et al. 2017 Gupta et al. This content is distributed under the terms of the Creative Commons Attribution 4.0 International license.

10.1128/mBio.01388-17.1 TEXT S1  Sequences used for construction of phylogenetic tree of bacterial P-type ATPase families. Download TEXT S1, DOCX file, 0.02 MB. Copyright © 2017 Gupta et al. 2017 Gupta et al. This content is distributed under the terms of the Creative Commons Attribution 4.0 International license.

**FIG 2:**
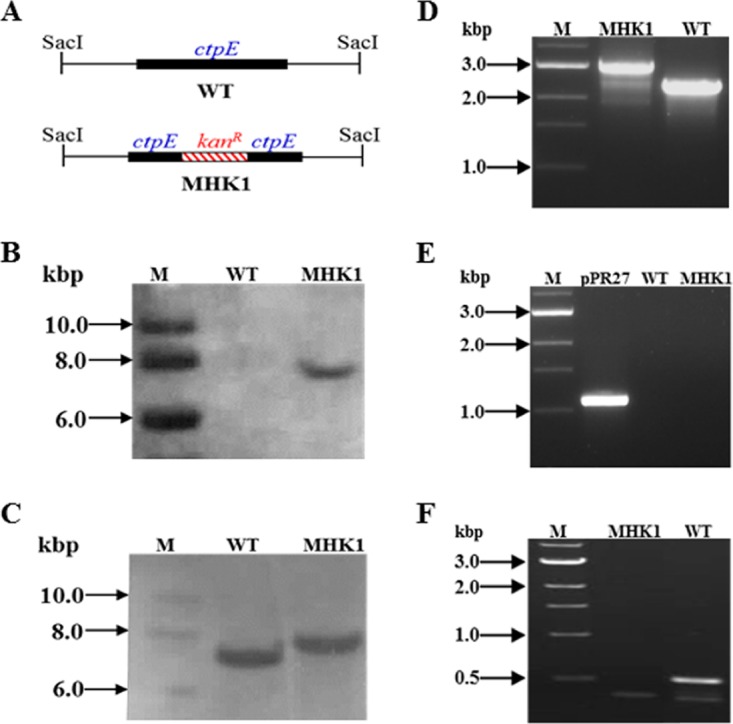
Confirmation of *ctpE* disruption in mutant MHK1. (A) Schematic representation of *ctpE* flanking region after SacI digestion from wild-type *Mycobacterium smegmatis* mc^2^155 (WT) and the mutant (MHK1), and the fragment sizes were 7.2 kb and 7.8 kb, respectively. (B and C) Genomic DNA from the WT and MHK1 strains was digested with SacI, and the Southern blot was developed by using probe for kanamycin cassette (B) or *ctpE* (C). Lanes M, DNA ladder. Amplification was carried out from genomic DNA isolated from the WT or MHK1 strain or plasmid pPR27. (D) The *ctpE* gene was amplified using primers HK11F and HK11R. The expected band was 2,996 bp from MHK1 and 2,367 bp from the WT. (E) The *sacB* gene was amplified using primers SACBF and SACBR, and the expected band was 1,127 bp from the plasmid control (pPR27) or single crossover mutant. The absence of amplification in strain MHK1 confirmed a double crossover mutant. (F) Amplification against the portion of *ctpE* deleted after digestion with SphI and NcoI in disruption construct using primers LOSSF and LOSSR. The expected band size was 611 bp, and the presence of this band was expected only in the WT.

**FIG 3:**
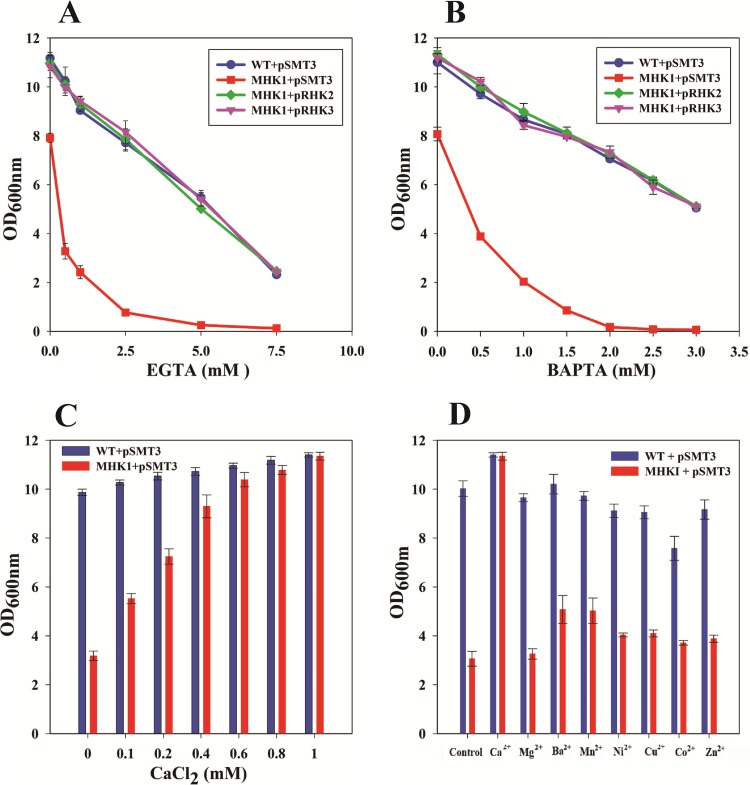
Growth defects of the MHK1 mutant and restoration by complementation or CaCl_2_. Wild-type *Mycobacterium smegmatis* mc^2^155 carrying pSMT3 (WT+pSMT3), the MHK1 mutant carrying pSMT3 (MHK1+pSMT3), mutant complemented with *M. smegmatis ctpE* (MHK1 carrying pRHK2 [MHK1+pRHK2]), and mutant complemented with *M. tuberculosis Rv0908* (MHK1+pRHK3) were grown in Sauton’s medium containing various concentrations of EGTA (A) and BAPTA (B). (C) Ca^2+^ is able to restore the growth of MHK1 in a dose-dependent manner. Strains were inoculated in Sauton’s medium containing 1.0 mM EGTA and 0.1 to 1.0 mM of Ca^2+^. (D) Growth of the MHK1 mutant was not restored by cations other than Ca^2+^. Strains were inoculated in Sauton’s medium containing 1.0 mM EGTA and 1.0 mM concentrations of different cations except Zn^2+^ and Co^2+^, which were used at 0.5 mM. Cell growth in these experiments was monitored after 48 h of incubation at 37°C by measuring absorbance or optical density at 600 nm. Experiments were performed three times; values are averages, and standard deviations are shown as error bars.

To ascertain the functional role of *ctpE*, the *ctpE* gene was disrupted by homologous recombination, and disruption of *ctpE* was confirmed by Southern hybridization and PCR amplifications (Fig. 2). The *ctpE-*disrupted strain was designated MHK1. The mutant showed normal growth in standard Luria-Bertani (LB) broth and Middlebrook 7H9 medium, but growth of the mutant was slow in Sauton’s medium, and culturing the mutant in Sauton’s medium could not achieve a final cell density similar to that of the wild type (WT) (routine observation). Most P-type ATPases are cation transporters; we screened strain MHK1 against increasing concentrations of various cations and chelators. The MHK1 strain was sensitive to Ca^2+^-specific chelators EGTA and 1,2-bis(*o*-aminophenoxy)ethane-*N*,*N*,*N*′,*N*′-tetraacetic acid (BAPTA) compared to the WT (Fig. 3A and B) but not to other chelators such as EDTA, *N*,*N*,*N*′,*N*′-tetrakis(2-pyridylmethyl)ethane-1,2-diamine (TPEN), or phenanthroline (Fig. S2). To determine whether the EGTA- and BAPTA-sensitive phenotype was due to specific scavenging of Ca^2+^ ions from the medium and not due to nonspecific chelation of other cations, we supplemented Sauton’s medium with different cations in the presence of 1.0 mM EGTA. Ca^2+^ was able to rescue the growth (Fig. 3C) of strain MHK1, and the growth defect was reversed in a dose-dependent manner (Fig. 3C). Equimolar concentrations of other cations such as Mg^2+^, Ba^2+^, Mn^2+^, Ni^2+^, and Cu^2+^ or 0.5 mM Co^2+^ or Zn^2+^ were not able to restore the growth of MHK1 (Fig. 3D).

10.1128/mBio.01388-17.3 FIG S2  Growth of *Mycobacterium smegmatis* mc^2^155 (WT) and the mutant (MHK1) in the presence of various metal ion chelators. Strains were grown in Luria-Bertani broth, and cell growth was monitored after 48 h of incubation at 37°C by measuring absorbance at 600 nm. (A) EDTA; (B) TPEN; (C) phenanthroline. Download FIG S2, TIF file, 4.6 MB. Copyright © 2017 Gupta et al. 2017 Gupta et al. This content is distributed under the terms of the Creative Commons Attribution 4.0 International license.

To confirm that the EGTA- or BAPTA-sensitive phenotype of strain MHK1 was due to disruption of *ctpE*, we complemented MHK1 with ectopic expression of *ctpE* under the control of the *hsp60* promoter. Complemented strain MHK1 carrying pRHK2 was completely restored for the EGTA- or BAPTA-sensitive phenotype (Fig. 3A and B). CtpE ortholog Rv0908 from pathogenic *Mycobacterium tuberculosis* has 79% identity at the protein level with CtpE from *M. smegmatis* and was expected to have a similar physiological function. Complementation of strain MHK1 with heterologous expression of *Rv0908* also restored the growth of MHK1 in the presence of EGTA or BAPTA similar to *ctpE* from *M. smegmatis* (Fig. 3A and B).

### CtpE is responsible for uptake of Ca^2+^ in *M. smegmatis.*

**FIG 4:**
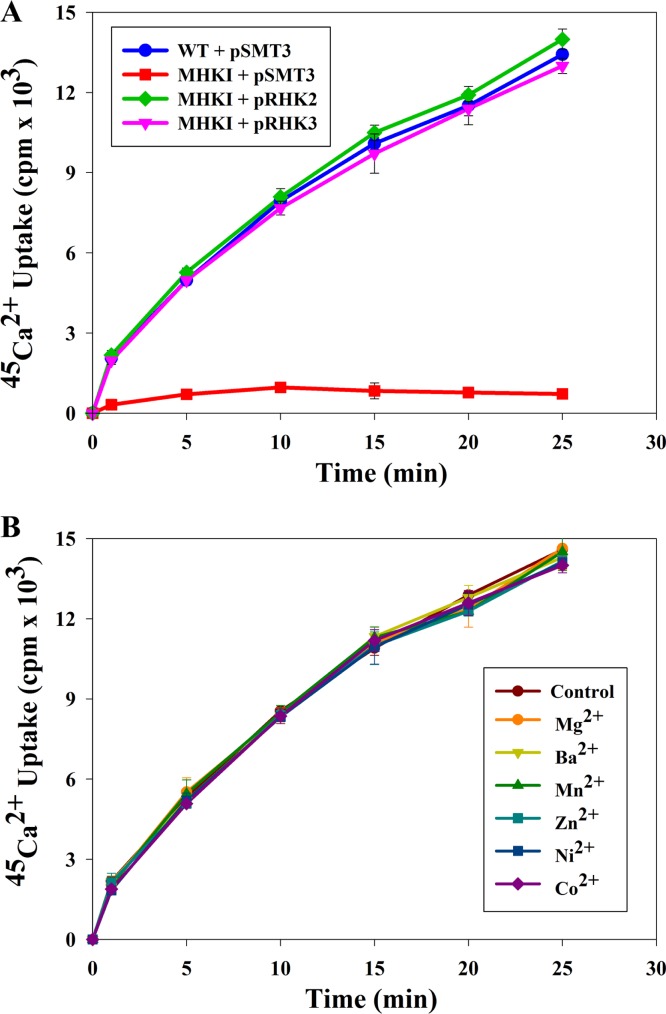
^45^Ca^2+^ uptake by *Mycobacterium smegmatis* strains. (A) *Mycobacterium smegmatis* mc^2^155 (WT strain carrying pSMT3 [WT+pSMT3]), the mutant (MHK1+pSMT3), mutant complemented with *M. smegmatis ctpE* (MHK1+pRHK2), and mutant complemented with *M. tuberculosis Rv0908* (MHK1+pRHK3) were grown to mid-log phase in Sauton’s medium supplemented with 1.0 mM EGTA, washed, and resuspended in assay buffer. ^45^Ca^2+^ uptake was initiated by adding 1 µCi/ml of ^45^Ca^2+^ and 10 mM CaCl_2_. Every 5 min, 1.0 ml of culture was taken out and subsequently passed through a 0.45-µm filter membrane. The membrane was washed with 1.0 mM EGTA containing HBSS buffer and dried in an oven. Radioactive counts were determined with a liquid scintillation counter. (B) Uptake of ^45^Ca^2+^ in *M. smegmatis* mc^2^155 was not inhibited by the indicated cations at 10 mM concentrations of Mg^2+^, Mn^2+^, Zn^2+^, Ni^2+^, and Co^2+^ or by 1.0 mM Ba^2+^ in the assay. Experiments were performed in triplicate; values are averages, and standard deviations are shown as error bars.

Sensitivity of MHK1 to Ca^2+^ chelators indicated that CtpE may be responsible for transport of Ca^2+^ into the cells. *M. smegmatis* strains were grown in Ca^2+^-deprived Sauton’s medium, and Ca^2+^ uptake was determined by using ^45^Ca^2+^ as a tracer (Fig. 4A). The accumulation of ^45^Ca^2+^ increased with time in the wild type, but there was no significant accumulation of ^45^Ca^2+^ in the MHK1 mutant strain. Expression of *ctpE* from *M. smegmatis* or its ortholog from *M. tuberculosis*, *Rv0908*, both restored the ^45^Ca^2+^ uptake in the mutant (Fig. 4A).

Cation selectivity of CtpE was examined by evaluating other cations as competitive inhibitors of Ca^2+^ uptake in the cells (Fig. 4B). Equimolar concentrations of MgCl_2_, MnCl_2_, ZnCl_2_, CoCl_2_, and NiCl_2_, or 1.0 mM BaCl_2_ (which precipitates at higher concentrations under assay conditions) did not compete for ^45^Ca^2+^ uptake, indicating that CtpE is a Ca^2+^-specific P-type ATPase.

### *ctpE* is transcribed as part of an operon.

**FIG 5:**
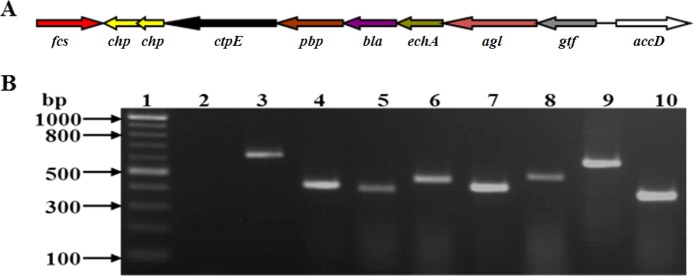
*ctpE* is expressed as a part of an operon. (A) Schematic representation of the genetic organization of the *M. smegmatis* mc^2^155 *ctpE* operon. The genes and the proteins they encode follow: *fcs*, feruloyl-CoA-synthetase; *chp*, conserved hypothetical protein; *ctpE*, cation-transporting P-type ATPase E; *pbp*, penicillin-binding protein 4; *bla*, metallo-β-lactamase; *echA*, enoyl-CoA-hydratase; *agl*, amylo-α-1,6-glucosidase; *gtf*, glycosyltransferase; *accD*, acyl-CoA-carboxylase. (B) RT-PCR analysis of the *Mycobacterium smegmatis* mc^2^155 *ctpE* operon. The intergenic regions between two genes were amplified. Lane 1, 100-bp DNA ladder; lane 2, RT negative control; lane 3, amplified product of region between *gtf* and *agl*; lane 4, region between *agl* and *echA*; lane 5, region between *echA* and *bla*; lane 6, region between *bla* and *pbp*; lane 7, region between *pbp* and *ctpE*; lane 8, region between *chp* and *chp*; lane 9, region between *ctpE* and *chp*; lane 10, *sigA* RT positive control.

Gene neighborhood analysis of *ctpE* revealed a putative operon with five upstream genes (*pbp* [penicillin-binding protein], *bla* [metallo-β-lactamase], *echA* [enoyl-coenzyme A {CoA} hydratase], *agl* [amylo-α-1,6-glucosidase], and *gtf* [glycosyltransferase]) and two downstream genes (*chp* [conserved hypothetical proteins]) in *M. smegmatis* (Fig. 5A). A similar gene organization was observed in other mycobacterial species (Fig. S3) with the exception that *agl* and *gtf* were absent at the same locus in other species. To confirm whether these eight genes existed in an operon in *M. smegmatis*, the boundaries of the putative operon intergenic regions were amplified by reverse transcriptase PCR (RT-PCR). Amplification of each junction between the genes confirmed that expression of these genes is transcriptionally coupled (Fig. 5B) and that they were transcribed as a polycistronic operon. This operon was designated the *ctpE* operon.

10.1128/mBio.01388-17.4 FIG S3  Genomic organization of *ctpE* orthologous gene in mycobacterial species. Gene names and the proteins encoded by the genes follow: *chp*, conserved hypothetical protein; *ctpE*, cation-transporting P-type ATPase E; *pbp*, penicillin-binding protein 4; *bla*, metallo-β-lactamase family protein; *echA*, enoyl-CoA-hydratase; *agl*, amylo-α-1,6-glucosidase; *gtf*, glycosyltransferase; *accD*, acetyl-CoA-carboxyl transferase. Download FIG S3, TIF file, 15.8 MB. Copyright © 2017 Gupta et al. 2017 Gupta et al. This content is distributed under the terms of the Creative Commons Attribution 4.0 International license.

### *ctpE* expression is regulated by Ca^2+^.

**FIG 6:**
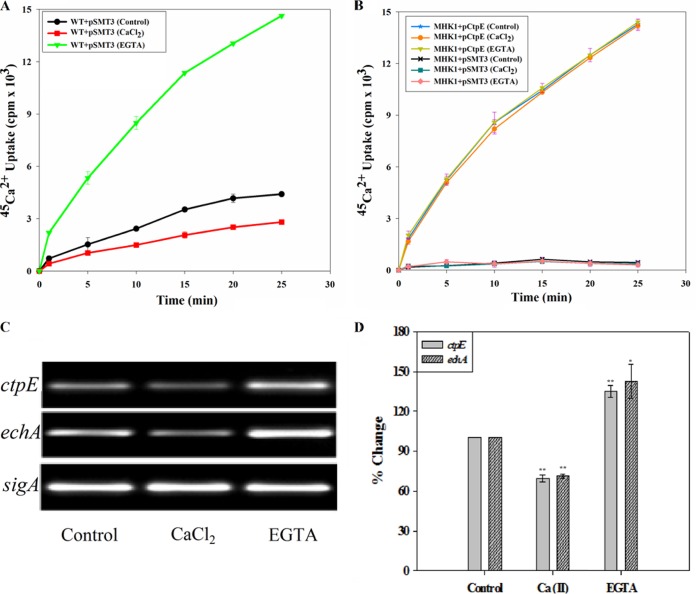
Ca^2+^ negatively regulates expression of *ctpE*. Role of Ca^2+^ on expression of *ctpE* in *Mycobacterium smegmatis* mc^2^155 strains. Strains were grown to mid-log phase in Sauton’s medium without any supplementation or supplemented with 1.0 mM EGTA or CaCl_2_ and used for uptake assays. (A and B) Uptake of ^45^Ca^2+^ in *M. smegmatis* mc^2^155 (WT) (A) and in the mutant (MHK1+pSMT3) and mutant complemented with *M. smegmatis ctpE* (MHK1+pRHK2) (B). (C) Semiquantitative reverse transcriptase PCR analysis of the *M. smegmatis* mc^2^155 *ctpE* and *echA*. *M. smegmatis* mc^2^155 was grown to mid-log phase in Sauton’s medium and treated with EGTA or CaCl_2_ for 2 h. RNA was isolated, and RT-PCR was carried out for *ctpE*, *echA*, and *sigA*. (D) Transcription profile of *ctpE* and *echA*. Quantification of amplified PCR product (Fig. 6C) was done by densitometry. *sigA* was taken as an endogenous control. The experiment was performed three times independently; values are averages, and standard deviations are shown as error bars. Values that are significantly different are indicated by asterisks as follows: *, *P* < 0.05; **, *P* < 0.01.

To check whether Ca^2+^ regulates expression of *ctpE*, we employed two approaches, ^45^Ca^2+^ uptake assays and semiquantitative reverse transcriptase PCR assay from cells grown in Ca^2+^-depleted or Ca^2+^-rich medium. First, Ca^2+^ uptake was monitored in WT cells grown in Ca^2+^-depleted or Ca^2+^-rich medium. ^45^Ca^2+^ accumulation increased fourfold in cells grown in Ca^2+^-depleted medium and decreased by nearly twofold in cells grown in Ca^2+^-rich medium compared to the control (cells grown in Sauton’s medium without any additives) (Fig. 6A). No such effects were observed on ^45^Ca^2+^ uptake in strain MHK1 expressing *ctpE* under the *hsp60* promoter (Fig. 6B), indicating that the influence of Ca^2+^ observed on ^45^Ca^2+^ uptake in WT cells is not due to posttranslational modifications of the CtpE protein. These results confirmed that Ca^2+^ deficiency induced the transcriptional expression of *ctpE*, resulting in increased uptake. Next, we checked the expression of *ctpE* and another gene in the operon, *echA*, in Ca^2+^-deficient or Ca^2+^-replenished growth conditions by semiquantitative RT-PCR. We observed significant increases in amplification of *ctpE* and *echA* from the RNA isolated from cells induced with Ca^2+^-deficient conditions and significant decreases in amplification of these two genes from RNA isolated from cells grown in Ca^2+^-replenished conditions in comparison to amplification from RNA isolated from cells grown in medium with no additives (Fig. 6C and D), indicating that the expression of *ctpE* was negatively regulated by the Ca^2+^ concentration in the medium. A similar level of expression and the Ca^2+^ influence on expression of *ctpE* and *echA* further confirmed the presence of both genes in a polycistronic mRNA and regulation of the operon by Ca^2+^.

### MHK1 shows cell envelope defects.

**FIG 7:**
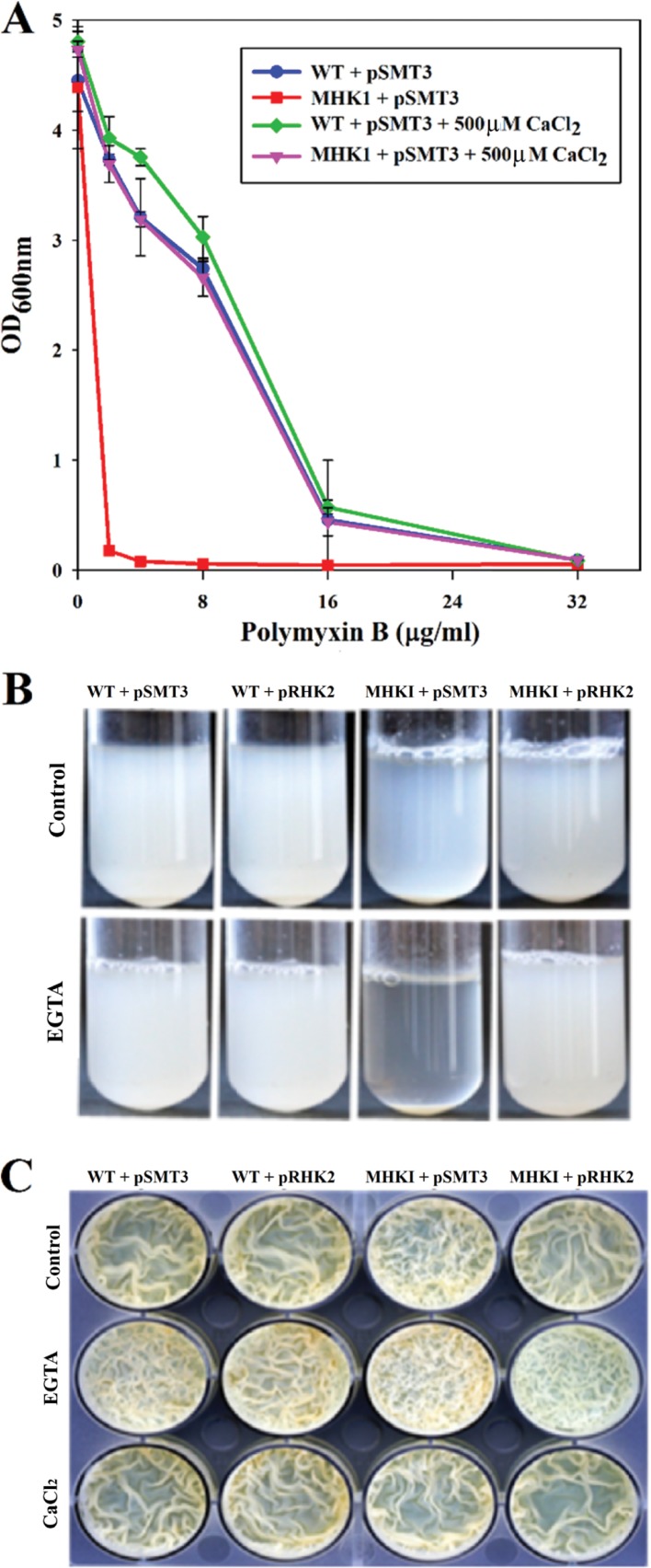
The MHK1 mutant shows defects in cell surface integrity. (A) The MHK1 mutant strain shows hypersensitivity to polymyxin B. The cultures were grown in Middlebrook 7H9 medium containing the indicated concentrations of polymyxin B and CaCl_2_. Cell growth was monitored after 72 h of incubation at 37°C by measuring absorbance at 600 nm. (B) MHK1 shows increased cell aggregation. All strains, *Mycobacterium smegmatis* mc^2^155 (WT+pSMT3), the mutant (MHK1+pSMT3), and mutant complemented with expression of *M. smegmatis ctpE* (MHK1+pRHK2), were grown in Middlebrook 7H9 medium up to mid-log phase and diluted in fresh medium with 1.0 mM EGTA or without EGTA. The cells were grown for up to 48 h at 37°C with shaking and left standing for 1.0 h. (C) MHK1 shows enhanced reticulation in biofilm formation. All the strains were grown in Sauton’s medium supplemented with 2.0% glucose in microtiter plates for 5 days at 37°C without any additive or with 1.0 mM EGTA or CaCl_2_.

We routinely observed that the MHK1 colonies were dry and rough in comparison to relatively oily and smooth colonies of the wild type on Ca^2+^-deficient medium. CtpE was found to transport Ca^2+^ in *M. smegmatis* and was part of an operon carrying genes with putative function in cell envelope biosynthesis or modification. This demanded an evaluation of the cell envelope defects in the MHK1 strain. We checked sensitivity and survival of MHK1 under various stress conditions to evaluate cell envelope defects and observed that the mutant did not differ significantly from the wild type in the presence of SDS, lysozyme, or cell wall-inhibiting antibiotics such as ethambutol or isoniazid (Fig. S4). Interestingly, MHK1 showed hypersensitivity to polymyxin B compared to the wild type (Fig. 7A) and also showed increased cellular aggregation (Fig. 7B) and increased reticulation in biofilm formation (Fig. 7C). These defects were enhanced by Ca^2+^ deficiency (1.0 mM EGTA) and were alleviated in the presence of excess Ca^2+^ (500 µM to 1.0 mM) in the growth medium. The phenotype of the complemented strain was similar to that of the wild type, indicating that CtpE-mediated Ca^2+^ uptake is important for the integrity of the cell envelope of *M. smegmatis* under Ca^2+^-deprived conditions.

10.1128/mBio.01388-17.5 FIG S4  Survival and antibiotic sensitivity assays to monitor cell surface defects in the MHK1 mutant. Cultures were grown to mid-log phase in Sauton’s medium containing 1.0 mM EGTA. (A and B) The cells were harvested and inoculated in fresh medium containing SDS (0.05% [wt/vol]) (A) and lysozyme (250 µg/ml) (B); cell survival was monitored every 2 h by plating the samples on Sauton’s medium agar plates and counting the colony-forming units. The results are expressed as percentage survival with respect to the cell count at time zero. (C and D) Antibiotic sensitivity of the cultures was tested in Middlebrook 7H9 medium containing 0.05% Tween 80 and different concentrations of isoniazid (C) or ethambutol (D). Cell growth was monitored after 72-h incubation at 37°C by measuring absorbance at 600 nm. Download FIG S4, TIF file, 8.4 MB. Copyright © 2017 Gupta et al. 2017 Gupta et al. This content is distributed under the terms of the Creative Commons Attribution 4.0 International license.

## DISCUSSION

In this work, we demonstrate that CtpE is a Ca^2+^ uptake transporter in *M. smegmatis*. To the best of our knowledge, this is the first Ca^2+^ uptake transporter belonging to the energy-dependent P-type ATPase superfamily reported from any organism. Distribution of CtpE orthologs in several bacterial divisions further indicates the importance and essential role of Ca^2+^ in the growth and physiology of prokaryotes. Orthologs of CtpE were identified from 290 strains and were absent in archaea and many other divisions in bacteria, indicating that this family of P-type ATPases has recently evolved for specific Ca^2+^ uptake required for essential physiological functions or survival of these species under low-Ca^2+^ growth conditions. We observed that strain MHK1 showed significant growth defect only in the presence of high concentrations of EGTA or BAPTA, which indicates that CtpE is required for acquisition of Ca^2+^ under Ca^2+^-deficient conditions and that *M. smegmatis* possesses yet uncharacterized alternate Ca^2+^ uptake mechanism(s) operating at higher Ca^2+^ concentrations.

We observed that *ctpE* is negatively regulated at the transcription level by Ca^2+^. This Ca^2+^-mediated regulation of *ctpE* may be due to a Ca^2+^-sensing/regulatory system in *M. smegmatis*. Eukaryotic calcium channels, plasma membrane calcium transporter (PMCA) and sarcoendoplasmic reticulum calcium transporter (SERCA) are known to be regulated at the transcription level by Ca^2+^-dependent signaling involving calmodulin and protein kinases (27). There are few examples of gene regulation by Ca^2+^-binding proteins in prokaryotes (7), but mechanisms regulating expression of Ca^2+^ channels or transporters are not known in prokaryotes. Bacteria are known to possess calmodulin-like and EF-hand proteins, which are speculated to play a role in Ca^2+^ storage and signaling. *M. smegmatis* has a calmodulin-like protein (CAMLP) with functional similarity to eukaryotic calmodulin (28). This protein was implicated to have a role in phospholipid metabolism (29), and CAMLP from *Mycobacterium tuberculosis* has recently been shown to be important during infection (30). A. Ca^2+^/calmodulin-dependent protein kinase (Cam-kinase) was also purified and characterized from *M. smegmatis* (31), but direct roles of these proteins in gene regulation in *M. smegmatis* remain unexplored. The presence of components of Ca^2+^-sensing (CAMLP) and regulatory (Cam-kinase) machinery and our observation of Ca^2+^-dependent regulation of *ctpE* in *M. smegmatis* tempt us to speculate on possible roles of CAMLP and Cam-kinase in *ctpE* regulation.

We observed that *ctpE* is part of an operon with genes predicted to be involved in membrane/cell wall biosynthesis or integrity in *M. smegmatis* and other mycobacterial species (see Fig. S3 in the supplemental material), indicating the requirement of Ca^2+^ in processes involving these proteins. Ca^2+^ is known to influence phospholipid metabolism and membrane structure (11). Ca^2+^ concentration changes and Ca^2+^/calmodulin-mediated regulation have been implicated in phospholipid biosynthesis and translocation of phospholipids between the monolayers of bilayer membranes (11). In *Pseudomonas aeruginosa*, a lytic transglycosylase (SltB1) and penicillin-binding protein (PBP2) form a complex in the presence of Ca^2+^ through an EF-hand motif of PBP2. This complex is required for peptidoglycan polymerization and integrity of the cell envelope (15). Sequence analysis using Prosite (32) did not reveal an EF-hand pattern in any of the proteins encoded by the *ctpE* operon, but we observed that amylo-α-1,6-glucosidase (AGL) possessed conserved DXDXDG motif known for Ca^2+^ binding (33). It will be interesting to validate possible Ca^2+^ binding to AGL and interactions of this protein with other proteins encoded by the CtpE operon. We observed several morphological changes, namely, colony surface, increased cell aggregation, denser biofilm, and polymyxin B sensitivity in the MHK1 strain, reflecting the influence of intracellular Ca^2+^ on the cell envelope composition and integrity in *M. smegmatis*. It is not difficult to speculate that other proteins encoded by the *ctpE* operon play a role in maintaining the cell envelope integrity through a Ca^2+^-influenced process, but the role of other Ca^2+^-regulated systems cannot be ruled out at this stage.

In conclusion, we demonstrate that CtpE of *M. smegmatis* and *M. tuberculosis* is a Ca^2+^ uptake transporter, which is essential for growth under Ca^2+^-deficient conditions. CtpE is expressed as a part of an operon and Ca^2+^ negatively regulates expression of this operon. Intracellular Ca^2+^ acquired by CtpE is important for cell wall integrity in *M. smegmatis*. Structural and functional comparison of CtpE with Ca^2+^ efflux pumps of family 2 will help in identifying molecular determinants responsible for directionality in the Ca^2+^-transporting P-type ATPases.

## MATERIALS AND METHODS

### Bacterial strains, plasmids, and culture media.

Bacterial strains and plasmids used in this study are listed in Table 1. For oligonucleotides, see Table S2 in the supplemental material. *Escherichia coli* strains were grown in Luria-Bertani (LB) broth and LB agar supplemented with appropriate antibiotics. *Mycobacterium smegmatis* mc^2^155 (wild-type [WT]) and mutant strains were grown on LB medium supplemented with 0.05% Tween 80 or Middlebrook 7H9 medium supplemented with albumin-dextrose-catalase (ADC) or oleic acid-albumin-dextrose-catalase (OADC) or Sauton’s medium (pH 7.5) supplemented with 0.05% Tween 80 as described below for the particular experimental procedure.

10.1128/mBio.01388-17.7 TABLE S2  Primers used in this study. Download TABLE S2, DOCX file, 0.02 MB. Copyright © 2017 Gupta et al. 2017 Gupta et al. This content is distributed under the terms of the Creative Commons Attribution 4.0 International license.

**TABLE 1:** Bacterial strains and plasmids used in this study

Strain or plasmid	Relevant characteristic(s)	Source or reference
*E. coli* DH10B	F^−^ *mcrA* Δ(*mrr-hsdRMS-mcrBC*) φ80d*lacZ*ΔM15 Δ*lacX74 deoR recA1 ara*Δ*139* (*ara leu*)*7697* *galU galK* λ^−^ *rpsL endA1 nupG*	Gibco-BRL
*M. smegmatis*		
mc^2^155	High-frequency transformation strain derived from wild-type *M. smegmatis* mc^2^-6	41
MHK1	*M. smegmatis* mc^2^155; *ctpE*::Km^r^	This work

Plasmids		
pET23a	*E. coli* expression vector; T7 promoter; His tag	Novagen
pPR27	*Mycobacterium-E. coli* shuttle vector; temperature-sensitive; *oriM sacB* Gent^r^	40
pUC4K	Kanamycin resistance cassette containing vector; Km^r^ Amp^r^	GE Healthcare
pSMT3	*E. coli*-*Mycobacterium* shuttle vector; *hsp60* promoter; Hyg^r^	44
pRHK1	*M. smegmatis ctpE* with insertion of kanamycin resistance cassette and cloned in pPR27; Km^r^ Gent^r^	This work
pRHK2	*M. smegmatis ctpE* cloned in pSMT3; Hyg^r^	This work
pRHK3	*M. tuberculosis Rv0908* cloned in pSMT3; Hyg^r^	This work

### Bioinformatic analysis.

Orthologs were predicted with OrtholugeDB (34) or by reciprocal best-hits method (35). Multiple sequence alignments of the CtpE sequence from *M. smegmatis* and orthologous sequences from other species was done by ClustalW, and a phylogenetic tree was constructed by neighbor-joining method using MEGA version 6.0 (36). Topology predictions were carried out by TMHMM (37), and the operon was predicted with the pattern/Markov chain-based bacterial operon and gene prediction program, fgenesB (38).

### Molecular biology methods and cloning.

DNA manipulations, such as PCR, restriction digestions, and ligations, were conducted using standard procedures (39). To generate expression clones under the control of the *hsp60* promoter, *ctpE* from *M. smegmatis* mc^2^155 (using primers MSEF and MSER [F stands for forward, and R stands for reverse]) and its homologue *Rv0908* from *M. tuberculosis* (using primers RVEF and RVER) were amplified, appropriately digested, and ligated into pSMT3 at the EcoRV or HindIII site to generate pRHK2 and pRHK3, respectively.

### Construction of *ctpE*-disrupted mutant.

The disruption mutant for *ctpE* in *M. smegmatis* mc^2^155 was generated by homologous recombination with sucrose counterselection (40). *ctpE* was amplified from *M. smegmatis* mc^2^155 genomic DNA by PCR using primers HK11F and HK11R. The amplified product was cloned into pET23a at the XbaI site, and the resultant plasmid was further digested with SphI and NcoI, which resulted in loss of the 611-bp fragment, causing disruption of the *ctpE*. The ends were blunt ended using T_4_ DNA polymerase. The kanamycin resistance cassette was excised as a PstI fragment from pUC4K and was made blunt ended. It was then ligated with pET23a containing disrupted *ctpE*. This kanamycin cassette-inserted *ctpE* was excised with XbaI and cloned into suicidal vector pPR27 at the XbaI site to generate plasmid pRHK1. The pRHK1 vector was electroporated into *M. smegmatis* mc^2^155 (41), and the transformants were allowed to grow at 30°C on LB agar plates containing kanamycin. A single colony of the transformant was inoculated into LB broth with kanamycin at 30°C for 24 h to allow recombination. The mutants were then selected at 42°C on LB agar plates supplemented with 10% sucrose and kanamycin. The loss of suicidal plasmid was confirmed by the absence of growth of the mutant on gentamicin-containing LB agar medium at 30°C.

### Southern hybridization.

Five micrograms of the genomic DNA was restriction digested with SacI, and Southern hybridization was performed as described previously (39). The kanamycin resistance cassette from pUC4K or a 771-bp fragment of *ctpE* (amplified using primers CESF and CESR) was used as a probe for confirmation of *ctpE* disruption in the MHK1 mutant. The labeling of the probe and hybridization and detection of hybridizing fragment were carried out according to the manufacturer’s instructions (NEBlot Phototope kit and Phototope-Star Detection kit; New England Biolabs Inc., USA).

### Restoration of MHK1 growth with cations.

To check the growth of strain MHK1 with different cations, starter cultures of *M. smegmatis* mc^2^155 and MHK1 were grown in Sauton’s medium supplemented with 0.05% Tween 80 for 24 h, diluted 1:100, and added to medium containing 1.0 mM EGTA. Restoration of growth with different cations was monitored with the addition of 0.1 to 1.0 mM concentrations of CaCl_2_, 1.0 mM MgCl_2_, BaCl_2_, MnCl_2_, NiCl_2_, or CuSO_4_, or 0.5 mM CoCl_2_ or ZnCl_2_. Cells were cultured for 48 h with shaking at 37°C, and growth was monitored at 600 nm.

### Complementation analysis.

*M. smegmatis* MHK1 and mc^2^155 were transformed with pSMT3, pRHK2, or pRHK3 by electroporation. Complementation was checked by growing all strains in Sauton’s medium supplemented with 0.05% Tween 80 and 50 µg ml^−1^ hygromycin with different concentrations of EGTA or 1,2-bis(*o*-aminophenoxy)ethane-*N*,*N*,*N*′,*N*′-tetraacetic acid (BAPTA) for 48 h, and the growth was monitored at 600 nm.

### Ca^2+^ uptake assays.

*M. smegmatis* strains were grown in Sauton’s medium without any additive or with 1.0 mM EGTA or 1.0 mM Ca^2+^. The cells were harvested, washed twice, and resuspended at a cell density of 1.0 OD_600_ (optical density at 600 nm) unit/ml in Sauton’s medium without any additive. The cells were incubated at 37°C for 15 min, and then ^45^Ca^2+^ uptake was initiated by the addition of ^45^Ca^2+^ to a final concentration of 1.0 μCi/ml and 10 mM cold carrier CaCl_2_. At the indicated time, 1.0-ml aliquots were filtered through 0.45-μm nitrocellulose filters, which were prewashed with 1.0 mM EGTA containing Hanks’ balanced salt solution (HBSS). The cells were washed twice with 1.0 mM EGTA containing HBSS, dried in an oven, and immersed in 1.0 ml of toluene-based scintillation fluid, and the amount of cell-accumulated ^45^Ca^2+^ was determined by liquid scintillation counting using a Wallac MicroBeta TriLux instrument (Perkin Elmer). Identical assay reaction mixtures were incubating on ice as controls. The counts from the ice controls were deducted from the counts of the test reaction mixtures incubated at 37°C.

### RNA isolation and reverse transcriptase PCR.

Total RNA from *M. smegmatis* mc^2^155 was isolated using an RNA isolation kit (Qiagen). The RNA was treated with DNase I and purified using an RNeasy kit (Qiagen). The absence of DNA contamination in RNA samples was confirmed by the absence of PCR amplification of *sigA* using SIGAF and SIGAR primers set by *Taq* polymerase.

To characterize the *ctpE* operon, approximately 400- to 600-bp junction regions between two genes were amplified with the help of junction primers listed in Table S2. Reverse transcription PCR was carried out using 1.0 μg total RNA by One-Step RT-PCR (reverse transcriptase PCR) kit (Qiagen). Reaction mixtures were incubated at 50°C for 30 min, followed by denaturation at 95°C for 15 min. This was followed by 30 cycles, with 1 cycle consisting of denaturation at 94°C for 30 s, annealing at (55°C to 65°C) for 1 min, and extension at 72°C for 30 to 40 s for different amplifications. The final extension was done at 72°C for 10 min. Amplification products were detected in 1.0% agarose gels along with the 100-bp ladder. For each sample, a reverse transcriptase-negative reaction was also performed to rule out DNA contamination. *sigA* was amplified as a positive control of RT-PCR using SIGAF and SIGAR primers.

To perform semiquantitative RT-PCR, the *M. smegmatis* mc^2^155 cultures were grown in Sauton’s medium until they reached an OD_600_ of 1.0. The cultures were then treated with 7.5 mM EGTA or 2.5 mM CaCl_2_ and allowed to grow for 2 h, and the total RNA was isolated using RNA isolation kit (Qiagen). Total RNA was quantified with a NanoDrop spectrophotometer (NanoDrop 1000; Thermo Scientific). Semiquantitative RT-PCR is carried out using 1.0 μg total RNA of each sample with a One-Step RT-PCR kit (Qiagen). The reaction mixture was incubated at 50°C for 30 min, followed by denaturation at 95°C for 15 min. The products were then amplified by 30 cycles, with 1 cycle consisting of denaturation at 94°C for 30 s, annealing (with JRT4F and JRT4R primers or ECHF and ECHR primers for *ctpE* and e*chA*, respectively) at 60°C for 1 min, and extension at 72°C for 30 s. The final extension was carried out at 72°C for 10 min. Amplification products were detected in 1.5% agarose gels along with a 100-bp ladder. *sigA* was used as an internal control and was amplified using sigsA2F and -A2R primers. Quantitation of amplified PCR product was done by densitometry using AlphaEaseFC 4.0. Densitometry values of *ctpE* and *echA* were normalized with internal control *sigA*. *P* values were calculated using GraphPad and indicated as follows: *, *P* < 0.05; **, *P* < 0.01. Experiments were performed three times independently.

### Antibiotic sensitivity assays.

The antibiotic sensitivity assays were performed by growing *M. smegmatis* strains in Middlebrook 7H9 medium supplemented with ADC for 24 h and then inoculating the cultures as 1:200 dilutions in fresh medium containing a range of antibiotic concentrations, followed by incubation at 37°C and 200 rpm for 72 h. Growth was monitored at 600 nm.

### Biofilm formation assays.

Biofilm formation was analyzed by the method of Ojha et al. (42) with small modifications. Briefly, 12-well cell culture plates containing Sauton’s medium supplemented with 4.0 ml of 2.0% glucose without any other additive or with 1.0 mM CaCl_2_ or 1.0 mM EGTA were inoculated with 40 µl of mid-log-phase culture of each strain. Plates were sealed with Parafilm and incubated undisturbed at 37°C, and biofilm formation was observed after 5 days.

### Aggregation assays.

Aggregation assays were performed by the method of Klepp et al. (43) with slight modifications. Briefly, the strains were grown in Middlebrook 7H9 medium up to mid-log phase, diluted 1:100, and added to fresh medium containing 1.0 mM EGTA or lacking EGTA. The cells were grown for 48 h at 37°C and 200 rpm and then left undisturbed for 1.0 h at room temperature and observed for cell aggregation.
