# Supplementary figures and images for: A Novel Calcium Uptake Transporter of Uncharacterized P-Type ATPase Family Supplies Calcium for Cell Surface Integrity in Mycobacterium smegmatis
Source: mBio. 2017 Sep 26;8(5):e01388-17. doi: 10.1128/mBio.01388-17 (PMC5615198; doi:10.1128/mBio.01388-17)

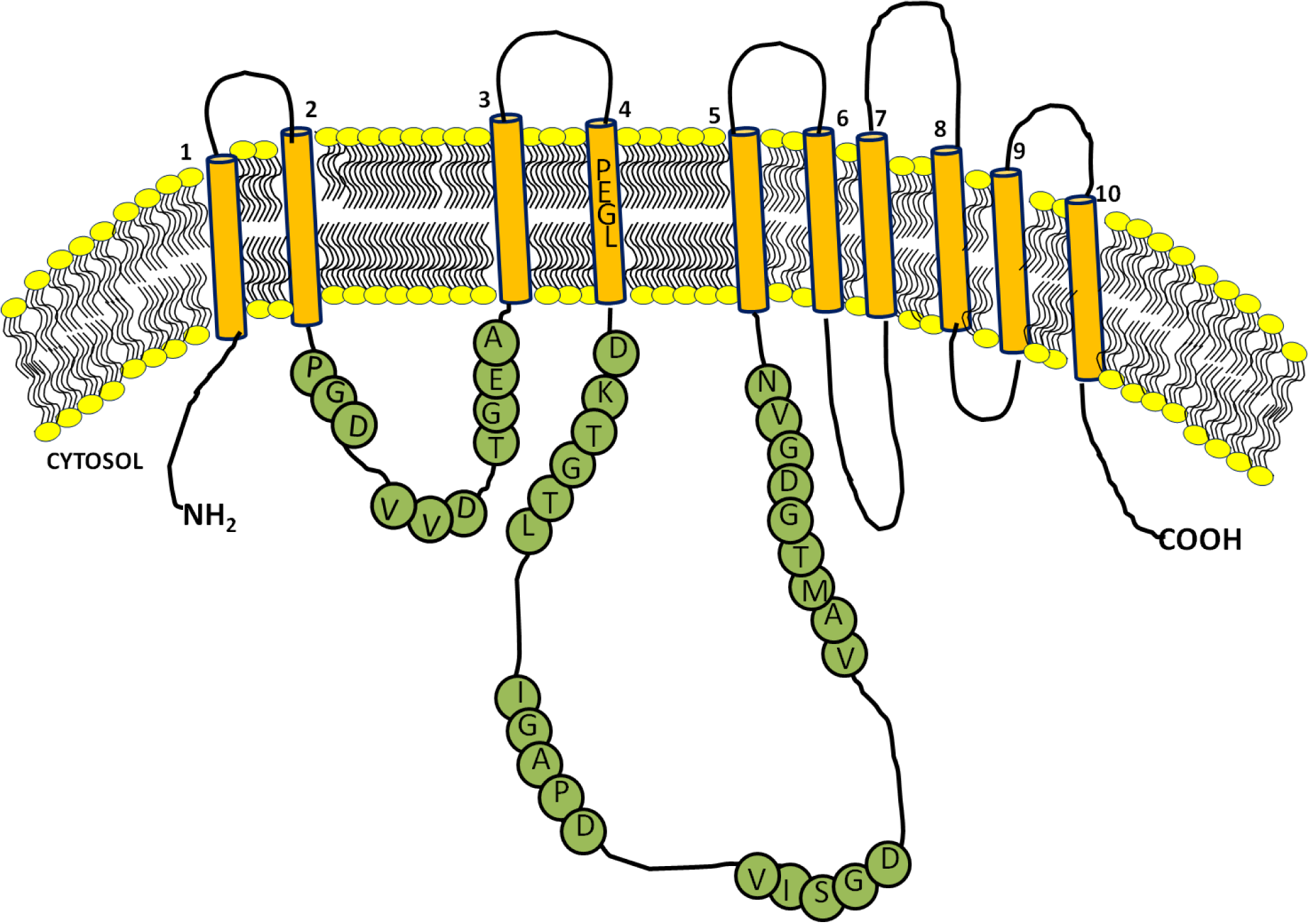

Supplement: FIG S1 [file mbo005173509sf1.tif]

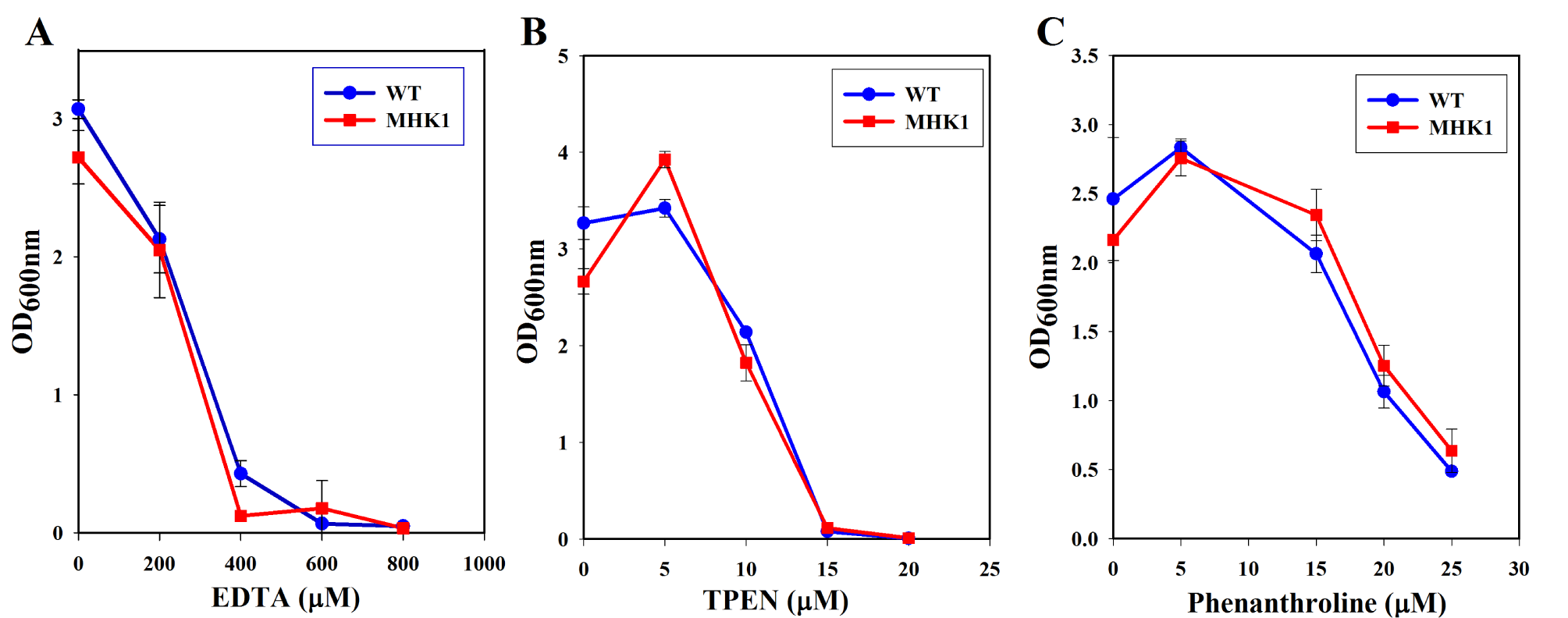

Supplement: FIG S2 [file mbo005173509sf2.tif]

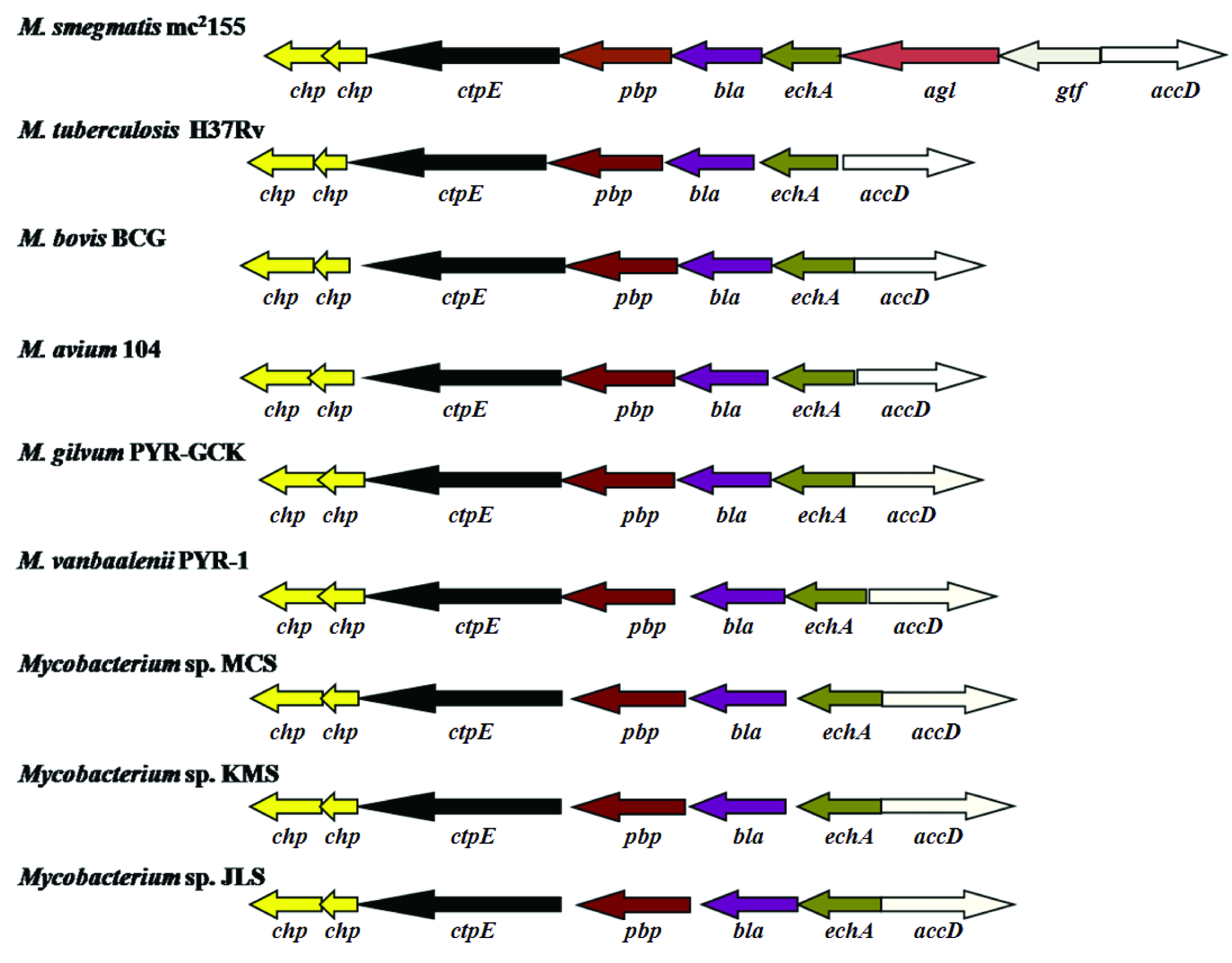

Supplement: FIG S3 [file mbo005173509sf3.tif]

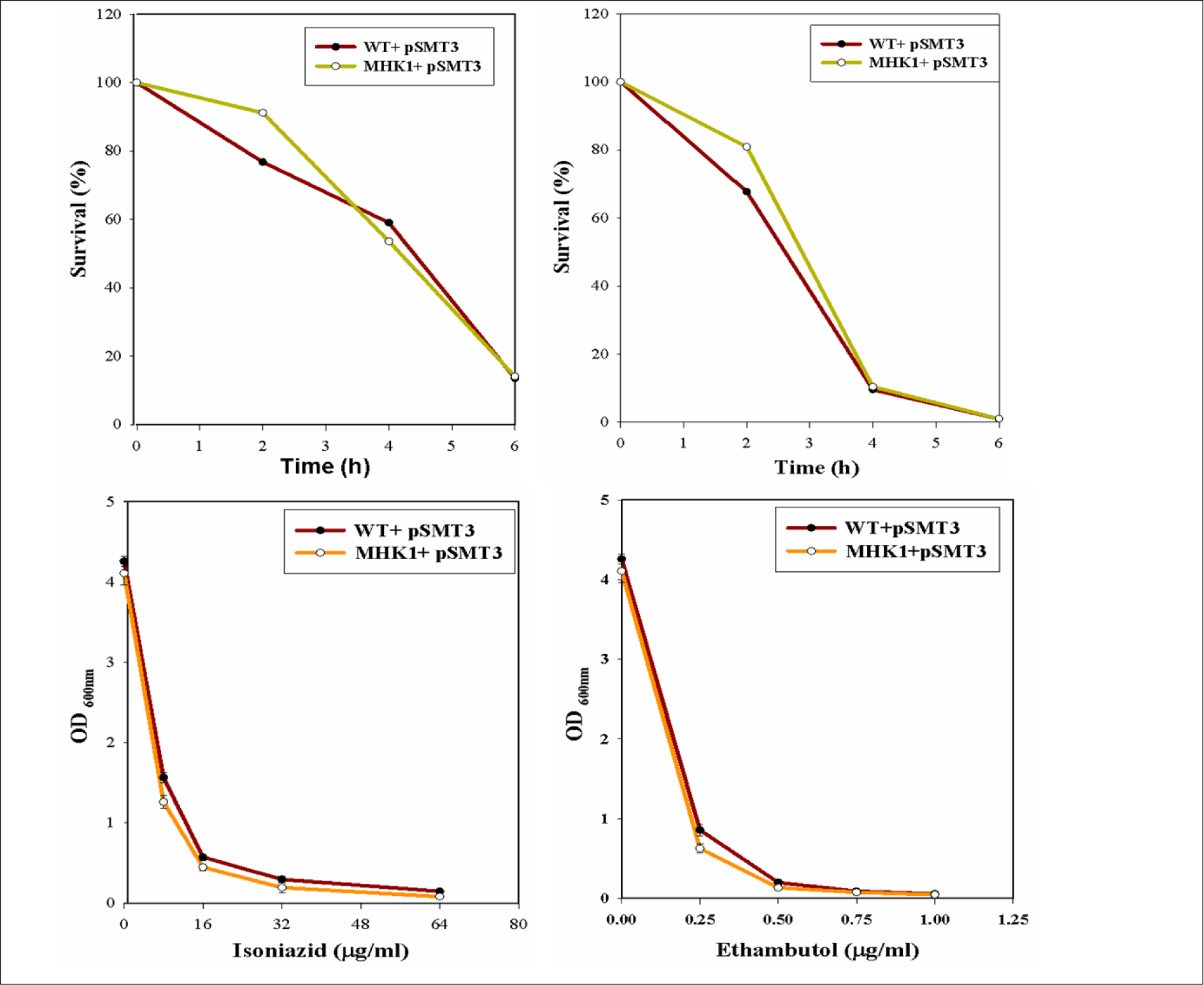

Supplement: FIG S4 [file mbo005173509sf4.tif]
