# Supplementary material for: A Novel Calcium Uptake Transporter of Uncharacterized P-Type ATPase Family Supplies Calcium for Cell Surface Integrity in Mycobacterium smegmatis
Source: mBio. 2017 Sep 26;8(5):e01388-17. doi: 10.1128/mBio.01388-17 (PMC5615198; doi:10.1128/mBio.01388-17)
Supplement: TABLE S1 [file mbo005173509st1.docx]

**Table S1. Orthologs of CtpE identified from various bacterial genomes.**

| S. No. | Name | Phylum | Locus Tag / Accession No. | GI |
| --- | --- | --- | --- | --- |
|  | *Acidothermus cellulolyticus* 11B | Actinobacteria | Acel_1545 | 117928752 |
|  | *Actinoplanes missouriensis* 431 | Actinobacteria | AMIS_18290 | 383776999 |
|  | *Arcanobacterium haemolyticum* DSM 20595 | Actinobacteria | Arch_1738 | 297572265 |
|  | *Arthrobacter aurescens* TC1 | Actinobacteria | AAur_3604 | 119963822 |
|  | *Arthrobacter chlorophenolicus* A6 | Actinobacteria | Achl_2077 | 220912829 |
|  | *Arthrobacter phenanthrenivorans* Sphe3 | Actinobacteria | Asphe3_20920 | 325963474 |
|  | *Arthrobacter sp.* FB24 | Actinobacteria | Arth_3471 | 116672013 |
|  | *Arthrobacter sp.* Rue61a | Actinobacteria | ARUE_c37480 | 403528778 |
|  | *Atopobium parvulum* DSM 20469 | Actinobacteria | Apar_0666 | 257784469 |
|  | *Beutenbergia cavernae* DSM 12333 | Actinobacteria | Bcav_1945 | 229820436 |
|  | *Bifidobacterium adolescentis* ATCC 15703 | Actinobacteria | BAD_1053 | 119026071 |
|  | *Bifidobacterium animalis* subsp. *animalis* ATCC 25527 | Actinobacteria | BANAN_05585 | 386867294 |
|  | *Bifidobacterium animalis* subsp. *lactis* AD011 | Actinobacteria | BLA_0713 | 219683199 |
|  | *Bifidobacterium animalis* subsp. *lactis* B420 | Actinobacteria | W7Y_1136 | 387821025 |
|  | *Bifidobacterium animalis* subsp. *lactis* BB-12 | Actinobacteria | BIF_00241 | 384189775 |
|  | *Bifidobacterium animalis* subsp. *lactis* Bi-07 | Actinobacteria | W91_1161 | 387822703 |
|  | *Bifidobacterium animalis* subsp. *lactis* Bl-04 | Actinobacteria | Balac_1135 | 241191159 |
|  | *Bifidobacterium animalis* subsp. *lactis* BLC1 | Actinobacteria | BLC1_1095 | 384194156 |
|  | *Bifidobacterium animalis* subsp. *lactis* CNCM I-2494 | Actinobacteria | BALAC2494_00107 | 384192564 |
|  | *Bifidobacterium animalis* subsp. *lactis* DSM 10140 | Actinobacteria | Balat_1135 | 241196565 |
|  | *Bifidobacterium animalis* subsp. *lactis* V9 | Actinobacteria | BalV_1099 | 384195721 |
|  | *Bifidobacterium bifidum* BGN4 | Actinobacteria | BBB_0603 | 390936517 |
|  | *Bifidobacterium bifidum* PRL2010 | Actinobacteria | BBPR_0619 | 311064027 |
|  | *Bifidobacterium bifidum* S17 | Actinobacteria | BBIF_0643 | 310287164 |
|  | *Bifidobacterium breve* ACS-071-V-Sch8b | Actinobacteria | HMPREF9228_0678 | 384196811 |
|  | *Bifidobacterium dentium* Bd1 | Actinobacteria | BDP_1482 | 283456342 |
|  | *Bifidobacterium longum* DJO10A | Actinobacteria | BLD_0263 | 189439126 |
|  | *Bifidobacterium longum* NCC2705 | Actinobacteria | BL1396 | 23465956 |
|  | *Bifidobacterium longum* subsp. *infantis* 157F | Actinobacteria | BLIF_1246 | 322689430 |
|  | *Bifidobacterium longum* subsp. *infantis* ATCC 15697 | Actinobacteria | Blon_0931 | 213691823 |
|  | *Bifidobacterium longum* subsp. *infantis* ATCC 15697 = JCM 1222 | Actinobacteria | BLIJ_0948 | 384198965 |
|  | *Bifidobacterium longum* subsp. *longum* JCM 1217 | Actinobacteria | BLLJ_1208 | 322691397 |
|  | *Bifidobacterium longum* subsp. *longum* JDM301 | Actinobacteria | BLJ_1204 | 296454342 |
|  | *Bifidobacterium longum* subsp. *longum* KACC 91563 | Actinobacteria | BLNIAS_01102 | 384201308 |
|  | *Catenulispora acidiphila* DSM 44928 | Actinobacteria | Caci_8836 | 256397926 |
|  | *Conexibacter woesei* DSM 14684 | Actinobacteria | Cwoe_1723 | 284043185 |
|  | *Coriobacterium glomerans* PW2 | Actinobacteria | Corgl_0850 | 328955445 |
|  | *Corynebacterium jeikeium* K411 | Actinobacteria | jk0273 | 68535338 |
|  | *Corynebacterium kroppenstedtii* DSM 44385 | Actinobacteria | ckrop_0452 | 237785067 |
|  | *Corynebacterium urealyticum* DSM 7109 | Actinobacteria | cur_1295 | 172040975 |
|  | *Corynebacterium variabile* DSM 44702 | Actinobacteria | CVAR_0643 | 340793605 |
|  | *Eggerthella lenta* DSM 2243 | Actinobacteria | Elen_1182 | 257790935 |
|  | *Frankia alni* ACN14a | Actinobacteria | FRAAL4310 | 111223709 |
|  | *Frankia sp.* CcI3 | Actinobacteria | Francci3_2796 | 86741483 |
|  | *Frankia sp.* EAN1pec | Actinobacteria | Franean1_2470 | 158314300 |
|  | *Frankia sp.* EuI1c | Actinobacteria | FraEuI1c_2712 | 312196552 |
|  | *Frankia* symbiont of *Datisca glomerata* | Actinobacteria | FsymDg_2857 | 336178733 |
|  | *Gardnerella vaginalis* 409-05 | Actinobacteria | HMPREF0424_0568 | 283783050 |
|  | *Gardnerella vaginalis* ATCC 14019 | Actinobacteria | HMPREF0421_20455 | 311114339 |
|  | *Gardnerella vaginalis* HMP9231 | Actinobacteria | HMPREF9231_1101 | 385802015 |
|  | *Geodermatophilus obscurus* DSM 43160 | Actinobacteria | Gobs_2789 | 284991236 |
|  | *Gordonia bronchialis* DSM 43247 | Actinobacteria | Gbro_1285 | 262201262 |
|  | *Gordonia polyisoprenivorans* VH2 | Actinobacteria | GPOL_c39050 | 378719393 |
|  | *Gordonia sp.* KTR9 | Actinobacteria | KTR9_1210 | 404213811 |
|  | *Intrasporangium calvum* DSM 43043 | Actinobacteria | Intca_0584 | 317123744 |
|  | *Kitasatospora setae* KM-6054 | Actinobacteria | KSE_44390 | 357391337 |
|  | *Kocuria rhizophila* DC2201 | Actinobacteria | KRH_18840 | 184201530 |
|  | *Leifsonia xyli* subsp. *xyli* str. CTCB07 | Actinobacteria | Lxx10430 | 50954725 |
|  | *Microbacterium testaceum* StLB037 | Actinobacteria | MTES_1807 | 323358255 |
|  | *Micrococcus luteus* NCTC 2665 | Actinobacteria | Mlut_20490 | 239918519 |
|  | *Modestobacter marinus* | Actinobacteria | MODMU_2716 | 389864390 |
|  | *Mycobacterium abscessus* ATCC 19977 | Actinobacteria | MAB_0962 | 169628059 |
|  | *Mycobacterium africanum* GM041182 | Actinobacteria | MAF_09170 | 339630972 |
|  | *Mycobacterium avium* 104 | Actinobacteria | MAV_1031 | 118464682 |
|  | *Mycobacterium avium* subsp. *paratuberculosis* K-10 | Actinobacteria | MAP0843 | 41406941 |
|  | *Mycobacterium bovis* AF2122/97 | Actinobacteria | Mb0932 | 31792096 |
|  | *Mycobacterium bovis* BCG str. Mexico | Actinobacteria | BCGMEX_0931 | 378770665 |
|  | *Mycobacterium bovis* BCG str. Pasteur 1173P2 | Actinobacteria | BCG_0960 | 121636831 |
|  | *Mycobacterium bovis* BCG str. Tokyo 172 | Actinobacteria | JTY_0930 | 224989302 |
|  | *Mycobacterium canettii* CIPT 140010059 | Actinobacteria | MCAN_09081 | 340625919 |
|  | *Mycobacterium chubuense* NBB4 | Actinobacteria | Mycch_4374 | 392418158 |
|  | *Mycobacterium gilvum* PYR-GCK | Actinobacteria | Mflv_1758 | 145222348 |
|  | *Mycobacterium intracellulare* ATCC 13950 | Actinobacteria | OCU_09050 | 379745625 |
|  | *Mycobacterium intracellulare* MOTT-02 | Actinobacteria | OCO_09000 | 379752913 |
|  | *Mycobacterium intracellulare* MOTT-64 | Actinobacteria | OCQ_09130 | 379760350 |
|  | *Mycobacterium marinum* M | Actinobacteria | MMAR_4622 | 183984589 |
|  | *Mycobacterium massiliense* str. GO 06 | Actinobacteria | MYCMA_0510 | 397678748 |
|  | *Mycobacterium rhodesiae* NBB3 | Actinobacteria | MycrhN_2685 | 375139832 |
|  | *Mycobacterium smegmatis* str. MC2 155 | Actinobacteria | MSMEI_5486 | 399989871 |
|  | *Mycobacterium sp.* JDM601 | Actinobacteria | JDM601_0830 | 333989470 |
|  | *Mycobacterium sp.* JLS | Actinobacteria | Mjls_4809 | 126437374 |
|  | *Mycobacterium sp.* KMS | Actinobacteria | Mkms_4515 | 119870544 |
|  | *Mycobacterium sp.* MCS | Actinobacteria | Mmcs_4428 | 108801391 |
|  | *Mycobacterium sp.* MOTT36Y | Actinobacteria | W7S_04435 | 387874293 |
|  | *Mycobacterium sp.* Spyr1 | Actinobacteria | Mspyr1_11420 | 315442781 |
|  | *Mycobacterium tuberculosis* CCDC5079 | Actinobacteria | CCDC5079_0839 | 385993964 |
|  | *Mycobacterium tuberculosis* CCDC5180 | Actinobacteria | CCDC5180_0831 | 385990365 |
|  | *Mycobacterium tuberculosis* CDC1551 | Actinobacteria | MT0931 | 15840327 |
|  | *Mycobacterium tuberculosis* CTRI-2 | Actinobacteria | MTCTRI2_0931 | 385997689 |
|  | *Mycobacterium tuberculosis* F11 | Actinobacteria | TBFG_10926 | 148822117 |
|  | *Mycobacterium tuberculosis* H37Ra | Actinobacteria | MRA_0915 | 148660686 |
|  | *Mycobacterium tuberculosis* H37Rv | Actinobacteria | RVBD_0908 | 397672726 |
|  | *Mycobacterium tuberculosis* H37Rv | Actinobacteria | Rv0908 | 15608048 |
|  | *Mycobacterium tuberculosis* KZN 1435 | Actinobacteria | TBMG_03081 | 253800068 |
|  | *Mycobacterium tuberculosis* KZN 4207 | Actinobacteria | TBSG_03101 | 375297302 |
|  | *Mycobacterium tuberculosis* KZN 605 | Actinobacteria | TBXG_003060 | 392433507 |
|  | *Mycobacterium tuberculosis* RGTB327 | Actinobacteria | MRGA327_05685 | 383306799 |
|  | *Mycobacterium tuberculosis* RGTB423 | Actinobacteria | MRGA423_05700 | 386003917 |
|  | *Mycobacterium tuberculosis* UT205 | Actinobacteria | UDA_0908 | 392385618 |
|  | *Mycobacterium ulcerans* Agy99 | Actinobacteria | MUL_0233 | 118616117 |
|  | *Mycobacterium vanbaalenii* PYR-1 | Actinobacteria | Mvan_4989 | 120405938 |
|  | *Nakamurella multipartita* DSM 44233 | Actinobacteria | Namu_2937 | 258653108 |
|  | *Nocardia cyriacigeorgica* GUH-2 | Actinobacteria | NOCYR_1892 | 379708113 |
|  | *Nocardia farcinica* IFM 10152 | Actinobacteria | nfa17840 | 54023752 |
|  | *Nocardiopsis alba* ATCC BAA-2165 | Actinobacteria | B005_4127 | 403511550 |
|  | *Nocardiopsis dassonvillei* subsp. *dassonvillei* DSM 43111 | Actinobacteria | Ndas_1536 | 297560497 |
|  | *Olsenella uli* DSM 7084 | Actinobacteria | Olsu_0852 | 302335638 |
|  | *Propionibacterium propionicum* F0230a | Actinobacteria | HMPREF9154_2064 | 397670735 |
|  | *Pseudonocardia dioxanivorans* CB1190 | Actinobacteria | Psed_0349 | 331694236 |
|  | *Renibacterium salmoninarum* ATCC 33209 | Actinobacteria | RSal33209_2394 | 163841129 |
|  | *Rhodococcus equi* 103S | Actinobacteria | REQ_10470 | 312138497 |
|  | *Rhodococcus erythropolis* PR4 | Actinobacteria | RER_45380 | 226308025 |
|  | *Rhodococcus jostii* RHA1 | Actinobacteria | RHA1_ro05035 | 111022002 |
|  | *Rhodococcus opacus* B4 | Actinobacteria | ROP_50990 | 226364509 |
|  | *Rothia dentocariosa* ATCC 17931 | Actinobacteria | HMPREF0733_11205 | 311112874 |
|  | *Rothia mucilaginosa* DY-18 | Actinobacteria | RMDY18_17860 | 283458796 |
|  | *Sanguibacter keddieii* DSM 10542 | Actinobacteria | Sked_16240 | 269794938 |
|  | *Streptomyces avermitilis* MA-4680 | Actinobacteria | SAV_3899 | 29830442 |
|  | *Streptomyces cattleya* NRRL 8057 = DSM 46488 | Actinobacteria | SCAT_2417 | 357400008 |
|  | *Streptomyces cattleya* NRRL 8057 = DSM 46488 | Actinobacteria | SCATT_24010 | 386356048 |
|  | *Streptomyces coelicolor* A3(2) | Actinobacteria | SCO4332 | 21222724 |
|  | *Streptomyces flavogriseus* ATCC 33331 | Actinobacteria | Sfla_2734 | 357411943 |
|  | *Streptomyces griseus* subsp. *griseus* NBRC 13350 | Actinobacteria | SGR_3167 | 182436960 |
|  | *Streptomyces hygroscopicus* subsp. *jinggangensis* 5008 | Actinobacteria | SHJG_4786 | 386840870 |
|  | *Streptomyces scabiei* 87.22 | Actinobacteria | SCAB_50941 | 290959497 |
|  | *Streptomyces sp.* SirexAA-E | Actinobacteria | SACTE_3770 | 345001302 |
|  | *Streptosporangium roseum* DSM 43021 | Actinobacteria | Sros_8943 | 271970122 |
|  | *Thermobifida fusca* YX | Actinobacteria | Tfu_1834 | 72162233 |
|  | *Tsukamurella paurometabola* DSM 20162 | Actinobacteria | Tpau_1222 | 296138949 |
|  | Candidate division TM7 single-cell isolate TM7c | Candidatus Saccharibacteria | WP_010164672.1 | 497850516 |
|  | “*Candidatus* Saccharimonas aalborgensis” | Candidatus Saccharibacteria | WP_015641348.1 | 505454246 |
|  | *Chlorobium sp.* GBChlB | Chlorobi | KER10871.1 | 662569752 |
|  | *Chloroherpeton thalassium* | Chlorobi | WP_012501200.1 | 501492764 |
|  | *Chloroherpeton* *thalassium* ATCC 35110 | Chlorobi | Ctha_2669 | 193216366 |
|  | *Sphaerobacter thermophilus* | Chloroflexi | WP_012873680.1 | 502637174 |
|  | *Sphaerobacter thermophilus* DSM 20745 | Chloroflexi | Sthe_3245 | 269929146 |
|  | *Cyanothece sp.* PCC 7425 | Cyanobacteria | Cyan7425_4133 | 220909497 |
|  | *Thermosynechococcus elongatus* BP-1 | Cyanobacteria | tll0153 | 22297697 |
|  | *Trichodesmium erythraeum* IMS101 | Cyanobacteria | Tery_3629 | 113477109 |
|  | *Acetobacterium woodii* DSM 1030 | Firmicutes | Awo_c26510 | 379012481 |
|  | *Butyrivibrio proteoclasticus* B316 | Firmicutes | bpr_I1401 | 302670760 |
|  | *Clostridiales genomosp*. BVAB3 str. UPII9-5 | Firmicutes | HMPREF0868_0033 | 289450011 |
|  | *Clostridium beijerinckii* NCIMB 8052 | Firmicutes | Cbei_5027 | 150019835 |
|  | *Clostridium botulinum* A str. ATCC 19397 | Firmicutes | CLB_1334 | 153931922 |
|  | *Clostridium botulinum* A str. ATCC 3502 | Firmicutes | CBO1306 | 148379286 |
|  | *Clostridium botulinum* A str. Hall | Firmicutes | CLC_1344 | 153937819 |
|  | *Clostridium botulinum* A2 str. Kyoto | Firmicutes | CLM_1465 | 226948573 |
|  | *Clostridium botulinum* A3 str. Loch Maree | Firmicutes | CLK_0748 | 170761400 |
|  | *Clostridium botulinum* B str. Eklund 17B | Firmicutes | CLL_A3468 | 187935469 |
|  | *Clostridium botulinum* B1 str. Okra | Firmicutes | CLD_3262 | 170755865 |
|  | *Clostridium botulinum* Ba4 str. 657 | Firmicutes | CLJ_B1353 | 237794595 |
|  | *Clostridium botulinum* BKT015925 | Firmicutes | CbC4_1631 | 331269813 |
|  | *Clostridium botulinum* E3 str. Alaska E43 | Firmicutes | CLH_3256 | 188588044 |
|  | *Clostridium botulinum* F str. Langeland | Firmicutes | CLI_1391 | 153938291 |
|  | *Clostridium botulinum* H04402 065 | Firmicutes | H04402_01377 | 387817584 |
|  | *Clostridium difficile* 630 | Firmicutes | CD1659 | 126699263 |
|  | *Clostridium difficile* BI1 | Firmicutes | CDBI1_08165 | 384360933 |
|  | *Clostridium difficile* CD196 | Firmicutes | CD196_1582 | 260683324 |
|  | *Clostridium difficile* R20291 | Firmicutes | CDR20291_1557 | 260686920 |
|  | *Clostridium lentocellum* DSM 5427 | Firmicutes | Clole_3304 | 326792373 |
|  | *Clostridium novyi* NT | Firmicutes | NT01CX_2343 | 118444358 |
|  | *Clostridium phytofermentans* ISDg | Firmicutes | Cphy_1566 | 160879711 |
|  | *Clostridium sp.* SY8519 | Firmicutes | CXIVA_02820 | 339441346 |
|  | *Enterococcus faecalis* 62 | Firmicutes | EF62_1711 | 384518366 |
|  | *Enterococcus faecalis* D32 | Firmicutes | EFD32_1069 | 397699648 |
|  | *Enterococcus faecalis* OG1RF | Firmicutes | OG1RF_11036 | 384513000 |
|  | *Enterococcus faecalis* V583 | Firmicutes | EF1268 | 29375837 |
|  | *Enterococcus faecium* Aus0004 | Firmicutes | EFAU004_01883 | 383329201 |
|  | *Enterococcus faecium* DO | Firmicutes | HMPREF0351_11845 | 389869028 |
|  | *Enterococcus hirae* ATCC 9790 | Firmicutes | EHR_09125 | 392989035 |
|  | *Erysipelothrix rhusiopathiae* str. Fujisawa | Firmicutes | ERH_1200 | 336066437 |
|  | *Ethanoligenens harbinense* YUAN-3 | Firmicutes | Ethha_1130 | 317132098 |
|  | *Eubacterium eligens* ATCC 27750 | Firmicutes | EUBELI_00508 | 238916451 |
|  | *Eubacterium limosum* KIST612 | Firmicutes | ELI_0915 | 310826533 |
|  | *Eubacterium rectale* ATCC 33656 | Firmicutes | EUBREC_2264 | 238924621 |
|  | *Lactobacillus acidophilus* 30SC | Firmicutes | LAC30SC_04525 | 325956590 |
|  | *Lactobacillus acidophilus* NCFM | Firmicutes | LBA0903 | 58337206 |
|  | *Lactobacillus amylovorus* GRL 1112 | Firmicutes | LA2_04710 | 315038137 |
|  | *Lactobacillus amylovorus* GRL1118 | Firmicutes | LAB52_04485 | 385817475 |
|  | *Lactobacillus brevis* ATCC 367 | Firmicutes | LVIS_0116 | 116332791 |
|  | *Lactobacillus buchneri* CD034 | Firmicutes | LBUCD034_2246 | 406027972 |
|  | *Lactobacillus buchneri* NRRL B-30929 | Firmicutes | Lbuc_2145 | 331702492 |
|  | *Lactobacillus casei* ATCC 334 | Firmicutes | LSEI_0749 | 116494293 |
|  | *Lactobacillus casei* BD-II | Firmicutes | LCBD_0826 | 385822523 |
|  | *Lactobacillus casei* BL23 | Firmicutes | LCABL_08160 | 191637631 |
|  | *Lactobacillus casei* LC2W | Firmicutes | LC2W_0826 | 385819357 |
|  | *Lactobacillus casei* str. Zhang | Firmicutes | LCAZH_0692 | 301065804 |
|  | *Lactobacillus crispatus* ST1 | Firmicutes | LCRIS_00956 | 295692818 |
|  | *Lactobacillus delbrueckii* subsp. *bulgaricus* 2038 | Firmicutes | LBU_0681 | 385815499 |
|  | *Lactobacillus delbrueckii* subsp. *bulgaricus* ATCC BAA-365 | Firmicutes | LBUL_0722 | 116513877 |
|  | *Lactobacillus delbrueckii* subsp. *bulgaricus* ND02 | Firmicutes | LDBND_0726 | 313123491 |
|  | *Lactobacillus gasseri* ATCC 33323 | Firmicutes | LGAS_0852 | 116629507 |
|  | *Lactobacillus helveticus* DPC 4571 | Firmicutes | lhv_0959 | 161507387 |
|  | *Lactobacillus helveticus* H10 | Firmicutes | LBHH_1214 | 385814007 |
|  | *Lactobacillus helveticus* R0052 | Firmicutes | R0052_07160 | 403515214 |
|  | *Lactobacillus johnsonii* DPC 6026 | Firmicutes | LJP_0840 | 385825774 |
|  | *Lactobacillus johnsonii* FI9785 | Firmicutes | FI9785_866 | 268319349 |
|  | *Lactobacillus johnsonii* NCC 533 | Firmicutes | LJ1400 | 42519270 |
|  | *Lactobacillus kefiranofaciens* ZW3 | Firmicutes | WANG_0763 | 336054273 |
|  | *Lactobacillus plantarum* JDM1 | Firmicutes | JDM1_0948 | 254556115 |
|  | *Lactobacillus plantarum* subsp. *plantarum* ST-III | Firmicutes | LPST_C0926 | 308180112 |
|  | *Lactobacillus plantarum* WCFS1 | Firmicutes | lp_1154 | 380032066 |
|  | *Lactobacillus rhamnosus* ATCC 8530 | Firmicutes | LRHK_731 | 385834647 |
|  | *Lactobacillus rhamnosus* GG | Firmicutes | LRHM_0713 | 385827433 |
|  | *Lactobacillus rhamnosus* GG | Firmicutes | LGG_00735 | 258507730 |
|  | *Lactobacillus rhamnosus* Lc 705 | Firmicutes | LC705_00728 | 258538919 |
|  | *Lactobacillus ruminis* ATCC 27782 | Firmicutes | LRC_05520 | 347525038 |
|  | *Lactobacillus salivarius* CECT 5713 | Firmicutes | HN6_00999 | 385840736 |
|  | *Lactobacillus salivarius* UCC118 | Firmicutes | LSL_1210 | 90962185 |
|  | *Lactobacillus sanfranciscensis* TMW 1.1304 | Firmicutes | LSA_02040 | 347533933 |
|  | *Lactococcus garvieae* ATCC 49156 | Firmicutes | LCGT_0104 | 347520710 |
|  | *Lactococcus garvieae* Lg2 | Firmicutes | LCGL_0104 | 385832073 |
|  | *Lactococcus lactis* subsp. *cremoris* A76 | Firmicutes | llh_10140 | 385838997 |
|  | *Lactococcus lactis* subsp. *cremoris* MG1363 | Firmicutes | llmg_0547 | 125623412 |
|  | *Lactococcus lactis* subsp. *cremoris* NZ9000 | Firmicutes | LLNZ_02810 | 389853737 |
|  | *Lactococcus lactis* subsp. *cremoris* SK11 | Firmicutes | LACR_0594 | 116511370 |
|  | *Lactococcus lactis* subsp. *lactis* CV56 | Firmicutes | CVCAS_0485 | 385830068 |
|  | *Lactococcus lactis* subsp. *lactis* Il1403 | Firmicutes | L168650 | 15672557 |
|  | *Lactococcus lactis* subsp. *lactis* KF147 | Firmicutes | LLKF_0554 | 281491042 |
|  | *Leuconostoc citreum* KM20 | Firmicutes | LCK_01012 | 170017364 |
|  | *Listeria ivanovii* subsp. *ivanovii* PAM 55 | Firmicutes | LIV_0659 | 347548135 |
|  | *Listeria seeligeri serovar* 1/2b str. SLCC3954 | Firmicutes | lse_0630 | 289433997 |
|  | *Melissococcus plutonius* ATCC 35311 | Firmicutes | MPTP_1579 | 332687042 |
|  | *Melissococcus plutonius* DAT561 | Firmicutes | MPD5_0470 | 379727050 |
|  | *Roseburia hominis* A2-183 | Firmicutes | RHOM_16610 | 347533595 |
|  | *Streptococcus gordonii* str. Challis substr. CH1 | Firmicutes | SGO_1458 | 157150720 |
|  | *Streptococcus infantarius* subsp. *infantarius* CJ18 | Firmicutes | Sinf_1414 | 379705735 |
|  | *Streptococcus intermedius* JTH08 | Firmicutes | SCIM_0485 | 392428376 |
|  | *Streptococcus mitis* B6 | Firmicutes | smi_0663 | 289167511 |
|  | *Streptococcus oralis* Uo5 | Firmicutes | SOR_0219 | 331265617 |
|  | *Streptococcus parasanguinis* ATCC 15912 | Firmicutes | HMPREF0833_10944 | 337282432 |
|  | *Streptococcus parasanguinis* FW213 | Firmicutes | Spaf_1522 | 387880006 |
|  | *Streptococcus pneumoniae* 670-6B | Firmicutes | SP670_1707 | 307127832 |
|  | *Streptococcus pneumoniae* 70585 | Firmicutes | SP70585_1664 | 225859379 |
|  | *Streptococcus pneumoniae* AP200 | Firmicutes | SPAP_1632 | 307068252 |
|  | *Streptococcus pneumoniae* ATCC 700669 | Firmicutes | SPN23F_16260 | 221232367 |
|  | *Streptococcus pneumoniae* CGSP14 | Firmicutes | SPCG_1598 | 182684568 |
|  | *Streptococcus pneumoniae* D39 | Firmicutes | SPD_1436 | 116515937 |
|  | *Streptococcus pneumoniae* G54 | Firmicutes | SPG_1535 | 194398153 |
|  | *Streptococcus pneumoniae* Hungary19A-6 | Firmicutes | SPH_1736 | 169834391 |
|  | *Streptococcus pneumoniae* INV104 | Firmicutes | INV104_13790 | 387626828 |
|  | *Streptococcus pneumoniae* INV200 | Firmicutes | SPNINV200_14470 | 387759737 |
|  | *Streptococcus pneumoniae* JJA | Firmicutes | SPJ_1518 | 225855053 |
|  | *Streptococcus pneumoniae* OXC141 | Firmicutes | SPNOXC_14260 | 387757856 |
|  | *Streptococcus pneumoniae* P1031 | Firmicutes | SPP_1644 | 225857242 |
|  | *Streptococcus pneumoniae* R6 | Firmicutes | spr1464 | 15903507 |
|  | *Streptococcus pneumoniae* ST556 | Firmicutes | MYY_1552 | 387788676 |
|  | *Streptococcus pneumoniae* Taiwan19F-14 | Firmicutes | SPT_1562 | 225861452 |
|  | *Streptococcus pneumoniae* TCH8431/19A | Firmicutes | HMPREF0837_11861 | 298503363 |
|  | *Streptococcus pneumoniae* TIGR4 | Firmicutes | SP_1623 | 15901459 |
|  | *Streptococcus pseudopneumoniae* IS7493 | Firmicutes | SPPN_07925 | 342164232 |
|  | *Streptococcus salivarius* 57.I | Firmicutes | Ssal_00450 | 387760719 |
|  | *Streptococcus salivarius* CCHSS3 | Firmicutes | SALIVB_1698 | 340399456 |
|  | *Streptococcus salivarius* JIM8777 | Firmicutes | SALIVA_1654 | 387784711 |
|  | *Streptococcus sanguinis* SK36 | Firmicutes | SSA_0712 | 125717565 |
|  | *Streptococcus suis* 05ZYH33 | Firmicutes | SSU05_1348 | 146319002 |
|  | *Streptococcus suis* 98HAH33 | Firmicutes | SSU98_1363 | 146321210 |
|  | *Streptococcus suis* A7 | Firmicutes | SSUA7_1192 | 386588453 |
|  | *Streptococcus suis* BM407 | Firmicutes | SSUBM407_0609 | 253755231 |
|  | *Streptococcus suis* D12 | Firmicutes | SSUD12_0607 | 386585774 |
|  | *Streptococcus suis* D9 | Firmicutes | SSUD9_0644 | 386583720 |
|  | *Streptococcus suis* GZ1 | Firmicutes | SSGZ1_1198 | 386578191 |
|  | *Streptococcus suis* JS14 | Firmicutes | SSUJS14_1310 | 386580243 |
|  | *Streptococcus suis* P1/7 | Firmicutes | SSU1180 | 253753894 |
|  | *Streptococcus suis* S735 | Firmicutes | YYK_05630 | 403061825 |
|  | *Streptococcus suis* SC84 | Firmicutes | SSUSC84_1213 | 253752069 |
|  | *Streptococcus suis* SS12 | Firmicutes | SSU12_1244 | 386582267 |
|  | *Streptococcus suis* ST1 | Firmicutes | SSUST1_0611 | 389856277 |
|  | *Streptococcus suis* ST3 | Firmicutes | SSUST3_0639 | 330832459 |
|  | *Streptococcus thermophilus* CNRZ1066 | Firmicutes | str1596 | 55823500 |
|  | *Streptococcus thermophilus* JIM 8232 | Firmicutes | STH8232_1837 | 386345292 |
|  | *Streptococcus thermophilus* LMD-9 | Firmicutes | STER_1558 | 116628287 |
|  | *Streptococcus thermophilus* LMG 18311 | Firmicutes | stu1596 | 55821572 |
|  | *Streptococcus thermophilus* MN-ZLW-002 | Firmicutes | Y1U_C1490 | 387910298 |
|  | *Streptococcus thermophilus* ND03 | Firmicutes | STND_1530 | 386087196 |
|  | *Gemmata obscuriglobus* | Planctomycetes | WP_033198365.1 | 702542924 |
|  | *Acidiphilium sp.* CAG:727 | Proteobacteria | WP_022419043.1 | 548198664 |
|  | *Burkholderia sp.* Ch1-1 | Proteobacteria | WP_007182804.1 | 494330642 |
|  | *Burkholderia thailandensis* | Proteobacteria | WP_006026792.1 | 492896386 |
|  | *Burkholderia thailandensis* | Proteobacteria | WP_009916359.1 | 497602175 |
|  | *Burkholderia thailandensis* | Proteobacteria | WP_015600848.1 | 505413746 |
|  | *Burkholderiales bacterium* GJ-E10 | Proteobacteria | BAP88149.1 | 723002995 |
|  | *Corallococcus sp.* CAG:1435 | Proteobacteria | WP_022060052.1 | 547329199 |
|  | *Corallococcus sp.* CAG:1435 | Proteobacteria | WP_022060244.1 | 547329400 |
|  | *Thiomonas sp.* FB-Cd | Proteobacteria | WP_031404387.1 | 670455588 |
|  | *Thiomonas sp.* FB-Cd | Proteobacteria | WP_031404403.1 | 670455604 |
|  | *Acholeplasma laidlawii* PG-8A | Tenericutes | ACL_0145 | 162447015 |
|  | Aster yellows witches'-broom phytoplasma AYWB | Tenericutes | AYWB_469 | 85057749 |
|  | “*Candidatus* Phytoplasma australiense” | Tenericutes | PAa_0673 | 197294707 |
|  | Onion yellows phytoplasma OY-M | Tenericutes | PAM_252 | 39938738 |
