# Supplementary material for: A Novel Calcium Uptake Transporter of Uncharacterized P-Type ATPase Family Supplies Calcium for Cell Surface Integrity in Mycobacterium smegmatis
Source: mBio. 2017 Sep 26;8(5):e01388-17. doi: 10.1128/mBio.01388-17 (PMC5615198; doi:10.1128/mBio.01388-17)
Supplement: TEXT S1 [file mbo005173509s1.docx]

**Sequences used for construction of phylogenetic tree are**: **Family 1 (Na^+^/K^+^):** *Leptospira biflexa* (WP_012389687.1), *Thiomicrospira crunogena* (WP_011370145.1), *Pelobacter carbinolicus* (WP_011342036.1), *Synechocystis sp.* PCC6803 (WP_010871930.1), *Thermomicrobium roseum* (ACM06351.1). **Family 2 (Ca^2+^ ):** *Mycobacterium smegmatis* (YP_888215), *Mycobacterium tuberculosis* (NP_216513.1), *Bacillus subtilis* (NP_389448), *Yersinia pestis* (WP_002209226), *Listeria monocytogenes* (NP_464367), *Streptococcus pneumoniae* (AAK75638 ), *Pseudomonas aeruginosa* (WP_003112435), *Ureaplasma parvum* (WP_012316988). **Family 3 (H^+^):** *Chlorobium tepidum* (WP_010933347.1), *Desulfotalea* *psychrophila* (WP_011188225.1), *Geobacter sulferreducens* (WP_010942986.1). **Family 4 (Mg^2+^):** *Leifsonia xyli* (WP_011186799.1), *E. coli* (WP_000471889.1), *Salmonella typhimurium* (ADX20223.1), *Bacteroides fragilis* (WP_005783820.1), *Mesoplasma florum* (WP_011183394.1). **Family 5 (Cu^2+^):** *M. smegmatis* (YP_889265), *M. tuberculosis* (NP_214606.1), *M. tuberculosis* (WP_009935939.1), *M. tuberculosis* (WP_003912985.1), *Anabaena variabilis* (WP_011320915.1), *E. coli* (NP_415017.1), *Staphylococcus aureus* (WP_000003267.1). **Family 6 (Heavy Metals):** *E. coli* (NP_417926.1), *M. smegmatis* (YP_889645), *M.* *tuberculosis* (NP_215985.1), *M. tuberculosis* (NP_216508.1), *M. tuberculosis* (NP_218260.1), *Bacillus cereus* (WP_000796568.1). **Family 7 (K^+/^KdpB):** *Escherichia coli* (NP_415225.1), *M. smegmatis* (YP_889634), *M. tuberculosis* (NP_215546.1), *Synechocystis sp.* (WP_010872556.1), *Bordetella* *bronchiseptica* (WP_010926987.1).**Family 23** *Acidothermus cellulolyticus* 11B (ABK53317.1), *Actinoplanes missouriensis* 431 (BAL87049.1), *Arcanobacterium haemolyticum* DSM 20595 (ADH93420.1), *Arthrobacter aurescens* TC1 (YP_949292.1), *Atopobium parvulum* DSM 20469 (ACV51095.1), *Beutenbergia cavernae* DSM 12333 (YP_002881962.1), *Bifidobacterium adolescentis* ATCC 15703 (BAF39834.1), *Catenulispora acidiphila* DSM 44928 (ACU77649.1), *Conexibacter woesei* DSM 14684 (ADB50150.1), *Coriobacterium glomerans* PW2 (AEB06963.1), *Corynebacterium jeikeium* K411 (CAI36425.1), *Eggerthella lenta* DSM 2243 (ACV55152.1), *Frankia alni* ACN14a (YP_714503.1), *Gardnerella vaginalis* 409-05 (ADB14669.1), *Geodermatophilus obscurus* DSM 43160 (ADB75419.1), *Gordonia bronchialis* DSM 43247 (ACY20577.1), *Intrasporangium calvum* DSM 43043 (ADU47129.1), *Kitasatospora setae* KM-6054 (BAJ30222.1), *Kocuria rhizophila* DC2201 (BAG30231.1), *Leifsonia xyli subsp. xyli* CTCB07 (AAT88908.1), *Microbacterium testaceum* StLB037 (YP_004224651.1), *Micrococcus* *luteus* NCTC 2665 (ACS31523.1), *Modestobacter marinus* (CCH88145.1), *Mycobacterium* *smegmatis* MC2 155 (YP_006570221.1), *Mycobacterium tuberculosis* H37Ra (ABQ72647.1), *Nakamurella* *multipartita* DSM 44233 (ACV79275.1), *Nocardia cyriacigeorgica* GUH-2 (YP_005263318.1), *Olsenella uli* DSM 7084 (ADK67965.1), *Propionibacterium propionicum* F0230a (AFN46693.1), *Pseudonocardia dioxanivorans* CB1190 (AEA22622.1), *Renibacterium salmoninarum* ATCC 33209 (ABY24120.1), *Rhodococcus equi* 103S (CBH47148.1), *Rothia dentocariosa* ATCC 17931 (ADP40662.1), *Sanguibacter keddieii* DSM 10542 (ACZ21559.1), *Streptomyces coelicolor* A3(2) (NP_628503.1), *Streptosporangium roseum* DSM 43021 (ACZ91575.1), *Thermobifida fusca* YX (AAZ55867.1), *Tsukamurella paurometabola* DSM 20162 (ADG77853.1), Candidate division TM7 single-cell isolate TM7c (WP_010164672.1), *Candidatus Saccharimonas aalborgensis* (WP_015641348.1), *Chlorobium sp*. GBChlB (KER10871.1), *Chloroherpeton thalassium* (WP_012501200.1), *Sphaerobacter thermophilus* (WP_012873680.1), *Cyanothece sp*. PCC 7425 (ACL46447.1), *Thermosynechococcus elongatus* BP-1 (BAC07706.1), *Trichodesmium* *erythraeum* IMS101 (YP_723170.1), *Acetobacterium woodii* DSM 1030 (AFA49404.1), *Butyrivibrio proteoclasticus* B316 (ADL34138.1), *Clostridiales genomosp.* BVAB3 str. UPII9-5 (ADC90983.1), *Enterococcus faecalis* 62 (YP_005705671.1), *Erysipelothrix rhusiopathiae* str. Fujisawa (BAK32254.1), *Ethanoligenens harbinense* YUAN-3 (ADU26681.1), *Eubacterium* *eligens* ATCC 27750 (ACR71521.1), *Lactobacillus acidophilus* 30SC (ADZ07063.1), *Leuconostoc citreum* KM20 (ACA82839.1), *Listeria ivanovii* subsp. *ivanovii* PAM 55 (CBW85137.1), *Melissococcus plutonius* ATCC 35311 (BAK22007.1), *Roseburia hominis* A2-183 (AEN98426.1), *Streptococcus gordonii* str. *Challis* substr. CH1 (ABV10197.1), *Gemmata* *obscuriglobus* (WP_033198365.1), *Acidiphilium sp.* CAG:727 (WP_022419043.1), *Burkholderia* *sp*. Ch1-1 (WP_007182804.1), *Corallococcus sp.* CAG:1435 (WP_022060052.1), *Thiomonas sp.* FB-Cd (WP_031404387.1), *Acholeplasma laidlawii* PG-8A (ABX80771.1), Aster yellows witches'-broom phytoplasma AYWB (ABC65586.1), Candidatus Phytoplasma australiense (YP_001799248.1), Onion yellows phytoplasma OY-M (BAD04337.1). **Family 24:** *Nocardia farcinica* (BAD57561), *M. tuberculosis* (WP_003911174.1), *M. tuberculosis* (WP_010886065.1), *Streptomyces coelicolor* (WP_011028388), *M. leprae* (NP_301578). **Family 25:** *Corynebacterium efficiens* (NP_738550), *Lactobacillus* *johnsonii* (WP_011162573), *Sinorhizobium meliloti* (AGG70318), *Thiomicrospira crunogena* (WP_011370145), *Streptomyces coelicolor* (NP_624500), **Family 26:** *Corynebacterium diphtheriae* (NP_938786), *Corynebacterium efficiens26* (NP_737067), *Corynebacterium glutamicum* (NP_599681). **Family 27:** *Caulobacter crescentus* (WP_010919283), *Agrobacterium fabrum* (WP_010971692.1), *Neisseria meningitidis* (WP_002219226.1), *Colwellia psychrerythraea* (WP_011042814.1), *Vibrio vulnificus* (WP_011080461.1). **Family 28:** *Fluoribacter dumoffii* (WP_010655002.1), *Legionella cherrii* (WP_028382517.1), *Legionella pneumophila* (WP_010945992.1). **Family 29:** *Bdellovibrio bacteriovorous* (CAE80402.1), *Flavobacterium phychrophilum* (WP_011963291.1), *Flavobacterium johnsoniae* (WP_012024602.1). **Family 30:** *Leptospira biflexa* (WP_012389404.1), *Bradyrhizobium* *diazoefficiens* (WP_011086596.1), *Burkholderia pseudomellei* (YP_111113.1), *Anabaena variabilis* (WP_011320775.1), *Flavobacterium johnsoniae* (WP_012026441.1). **Family 31:** *Methylococcus* *capsulatus* (WP_010961163.1). **Family 32:** *Treponema denticola* (WP_002681568.1), *Nostoc sp.* PCC712032 (BAB73703.1), *Thermosynechococcus elongates* (WP_011058044.1), *Aromatoleum* *aromaticum* (WP_011236174.1), Desulfovibrio *vulgaris* (WP_010940588.1).
