# Supplementary material for: A Novel Calcium Uptake Transporter of Uncharacterized P-Type ATPase Family Supplies Calcium for Cell Surface Integrity in Mycobacterium smegmatis
Source: mBio. 2017 Sep 26;8(5):e01388-17. doi: 10.1128/mBio.01388-17 (PMC5615198; doi:10.1128/mBio.01388-17)
Supplement: TABLE S2 [file mbo005173509st2.docx]

**Table S2**. Primers used in this study.

| **Primers** |  |  |
| --- | --- | --- |
| HK11F | TCC TCT AGA ATG GCT GCC GCC GGC CTC ACC GAT | This work |
| HK11R | CGA TCT AGA CTA TCT CCA CAC GCG CCG TTC TTC | This work |
| MSEF | GAT ATC AAT GGC TGC CGC CGG CCT CAC CGA T | This work |
| MSER | GAT ATC CTA TCT CCA CAC GCG CGT TCT TC | This work |
| RVEF | CCC AAG CTT AAT GAC CCG TTC GGC TTC GGC GAC AGC CGG TTT G | This work |
| RVER | CCC AAG CTT TTA TCG CCA CAC TCT CGC TTT CAC ACC GAG CAT | This work |
| CESF | CTG GCC CTG AAG GAC TCC GAC ATC | This work |
| CESR | CTA TCT CCA CAC GCG CCG TTC TTC | This work |
| JRT1F | CAT GCT GGA TCC CAC CAA TAT GAA | This work |
| JRT1R | CAC CGT TGA GTG TGA GCG CCT GCG | This work |
| JRT2F | TGT GGG CGT GTA CCG CAA CGA CTA | This work |
| JRT2R | GAT GAT GCC GAT CGC GCT GTT GGC | This work |
| JRT3F | GAC AAG GCC AAA GAC CTC TTG TCG | This work |
| JRT4F | CCG AGG CCT ACC AGA CGC | This work |
| JRT4R | CAT CGC GAC CGT GTG CCC | This work |
| JRT5F | GCC GGG GTG CAG GTC GCG | This work |
| JRT5R | ACT CGA CAT CGC CGT TGC | This work |
| JRT6F | AGA AGT GGG CGG CCG AGA | This work |
| JRT6R | TTC GCG GTC CAG GTT GAT | This work |
| JRT7F | CTG CTG CTG GTG CGC GCC | This work |
| JRT7R | GTC GGG CAG TTC GGC GGC | This work |
| JRT8F | TGC GAT GTG CCC GAG GAA | This work |
| JRT8R | GAA CAG GTG CCC GAG GAA | This work |
| SIGAF | ACC TTG AGG TGA CCG ACG ATC | This work |
| SIGAR | AGC TTC TGC GTG GCG TAG AG | This work |
| ECHF | CAA CCG CAT CGG GAC CCT | This work |
| ECHR | TTC GTA GGC GCC GTC GTC | This work |
| SIGA2F | CCA AGG GCT ACA AGT TCT CG | This work |
| SIGA2R | CTT GTT GAT CAC CTC GAC CA | This work |
| SACBF | TGA ACA GCA AAA AAA TGA AAA | This work |
| SACBR | GCT TGT AAT CAC CAC GAC ATA | This work |
| LOSSF | TGT CAG CGA TCT CAA GTC | This work |
| LOSSR | GTC TCG CCG TGC AAA CCC | This work |
